# *In silico* proteomic and phylogenetic analysis of the outer membrane protein repertoire of gastric *Helicobacter* species

**DOI:** 10.1038/s41598-018-32476-1

**Published:** 2018-10-18

**Authors:** Eva Bauwens, Myrthe Joosten, Joemar Taganna, Mirko Rossi, Ayla Debraekeleer, Alfred Tay, Fanny Peters, Steffen Backert, James Fox, Richard Ducatelle, Han Remaut, Freddy Haesebrouck, Annemieke Smet

**Affiliations:** 1 Department of Pathology, Bacteriology and Avian Diseases, Faculty of Veterinary Medicine, Ghent University, Merelbeke, Belgium; 2 Laboratory of Structural and Molecular Microbiology, Structural Biology Research Center, Flemish Institute for Biotechnology (VIB), Brussels, Belgium; 3 Structural Biology Brussels, Free University of Brussels (VUB), Brussels, Belgium; 4 Department of Food Hygiene and Environmental Health, Faculty of Veterinary Medicine, University of Helsinki, Helsinki, Finland; 5 The Marshall Centre for Infectious Diseases Research and Training, School of Pathology and Laboratory Medicine, University of Western Australia, Nedlands, Perth, Western Australia Australia; 6 University Erlangen Nürnberg, Department Biology, Division Microbiology, Erlangen, Germany; 7 Division of Comparative Medicine, Massachusetts Institute of Technology, Cambridge, MA USA; 8 Laboratorium of Experimental Medicine and Pediatrics, Faculty of Medicine and Health Sciences, University of Antwerp, Antwerp, Belgium

## Abstract

*Helicobacter (H*.*) pylori* is an important risk factor for gastric malignancies worldwide. Its outer membrane proteome takes an important role in colonization of the human gastric mucosa. However, in zoonotic non-*H*. *pylori* helicobacters (NHPHs) also associated with human gastric disease, the composition of the outer membrane (OM) proteome and its relative contribution to disease remain largely unknown. By means of a comprehensive survey of the diversity and distribution of predicted outer membrane proteins (OMPs) identified in all known gastric *Helicobacter* species with fully annotated genome sequences, we found genus- and species-specific families known or thought to be implicated in virulence. Hop adhesins, part of the *Helicobacter*-specific family 13 (*Hop*, *Hor* and *Hom*) were restricted to the gastric species *H*. *pylori*, *H*. *cetorum* and *H*. *acinonychis*. Hof proteins (family 33) were putative adhesins with predicted Occ- or MOMP-family like 18-stranded β-barrels. They were found to be widespread amongst all gastric *Helicobacter* species only sporadically detected in enterohepatic *Helicobacter* species. These latter are other members within the genus *Helicobacter*, although ecologically and genetically distinct. LpxR, a lipopolysaccharide remodeling factor, was also detected in all gastric *Helicobacter* species but lacking as well from the enterohepatic species *H*. *cinaedi*, *H*. *equorum* and *H*. *hepaticus*. In conclusion, our systemic survey of *Helicobacter* OMPs points to species and infection-site specific members that are interesting candidates for future virulence and colonization studies.

## Introduction

*H*. *pylori* colonizes the stomach of more than 50% of the human population. Infection with this microbe is considered to be the most important risk factor for gastric malignancies worldwide^1,2^. In contrast to most other Gram-negative bacteria, the outer membrane of *H*. *pylori* is equipped with a remarkably large set of OMPs^3^. There are approximately 64 well-annotated OMP genes present in *H*. *pylori*, encompassing 4% of its entire genome^4^. This unusually large set of predicted OMPs possibly originates as an adaptation of the gastric bacterium to the hostile environment of the stomach. The *H*. *pylori* OMPs can be divided into 6 groups: the Hop (*H*. *pylori* OMP), Hor (Hop-related), Hof (*Helicobacter* OMP), Hom (*Helicobacter* outer membrane), iron-regulated OMPs and efflux pump OMPs. Most of the *H*. *pylori* OMPs belonging to the Hop proteins, are predicted to function as porins or as adhesins that promote binding of the bacterium to the gastric mucosa^3,5^. More specifically, BabA (HopS), SabA (HopP), OipA (HopH), AlpA (HopC), AlpB (HopB), LabA (HopD), HopQ and HopZ are the best characterized *H*. *pylori* Hop adhesins that play a role in its colonization process^6–13^.

Besides *H*. *pylori*, also non-*H*. *pylori Helicobacter* (NHPH) species have been associated with gastric disease in humans, including gastritis, gastric and duodenal ulcers and mucosa-associated lymphoid tissue (MALT) lymphoma. Such NHPHs mostly originate from domestic animals, such as *H*. *suis* from pigs and *H*. *heilmannii*, *H*. *bizzozeronii*, *H*. *felis*, *H*. *salomonis*, *H*. *cynogastricus*, *H*. *baculiformis* and *H*. *ailurogastricus* from cats and dogs^14^. Recent comparative genomic studies highlighted that gastric NHPHs lack all known *H*. *pylori* adhesins described so far^15–18^. Furthermore, they share only few homologs of the Hor and Hom family. Only genes encoding homologs of the *H*. *pylori* Hof protein family are present in these animal-associated helicobacters, but in contrast to *H*. *pylori*, their *hof* genes are located in a ~10-kb locus^15^. For *H*. *heilmannii*, it has recently been shown that HofE and HofF play a key role in adhesion to the gastric mucosa, albeit with a higher affinity for gastric epithelial cells than for mucins^19^.

Based on the available data, it is clear that gastric NHPH species harbor a different OMP repertoire compared to *H*. *pylori* and that the mechanisms by which these NHPHs colonize the gastric mucosa remains largely unknown. Besides the domestic animal-related gastric *Helicobacter* species, also other gastric helicobacters, including *H*. *acynonichis* from wild felines, *H*. *cetorum* from marine mamals and *H*. *mustelae* from ferrets, with unknown OMP repertoire have been described.

Therefore, in the present study, we screened the genomes *in silico* from all gastric *Helicobacter* species known so far, for the presence of genes encoding putative OMPs. The predicted OMPs were then classified into families based on their protein sequence homology, using 3 different protein databases. Finally, phylogenetic analyses of the OMP sequences were applied to study the predicted functional relationships among the OMPs and to unravel the OMPs that could play a role in the colonization and virulence properties of gastric NHPHs. In addition, the genomes of 4 enterohepatic *Helicobacter* species and 2 closely-related *Campylobacter (C*.*)* species were included in the proteomic and phylogenetic analyses for comparison. These species are phylogenetically close to gastric *Helicobacter* species, yet are ecologically distinct in thriving on the mucosal surfaces of the intestinal tract and/or the liver, so that genomic comparison may point to niche-dependent genetic markers in the OM proteomes^20,21^. As a reference for functional annotation, we included the genome of *Escherichia (E*.*) coli*, for which the biological functions of the majority of its OMPs are well characterized.

Accordingly, this study identified several unique OMP families that are possibly important for colonization and virulence, in gastric *Helicobacter* species.

## Results

### Identification of OMP families

Fifty four genomes were analysed for candidate OMPs by screening their open reading frames for proteins with signal peptide and β-strand propensities using the HHomp tool^22^. A total of 3380 putative OMPs were identified among the different strains of *E*. *coli* (500) and the genera *Campylobacter* (141) and *Helicobacter* (2739). An overview of the number of OMPs per species and per strain is presented in Table 1. For the *Campylobacter* and the *E*. *coli* strains, the total number of OMPs per genome ranged between 21 and 27, and between 88 and 116, respectively. The mean number of OMPs was lower for the 4 enterohepatic helicobacters (a mean of 33 OMPs) with a minimum of 24 OMPs for *H*. *equorum* eqF1 and a maximum of 55 OMPs for *H*. *trogontum* R3554), compared to the gastric *Helicobacter* species (a mean of 66 OMPs) with a minimum of 47 OMPs for *H*. *salomonis* M45 and a maximum of 118 OMPs for *H*. *cetorum* MIT 00–7128). *H*. *mustelae*, which is able to colonize both the gastric and intestinal environment^23^, contained 49 OMPs (Table 1). Subsequently, these OMPs were classified into families based on protein sequence homology. From the 3380 OMPs, 2794 proteins could be classified into one of the 90 families known from the OMPdb reference database (OMPdb.org). The results are listed in Supplementary Table S1. For each of the 90 OMP families, the name and function is shown as well as the OMPs from the *E*. *coli*, *Campylobacter* and *Helicobacter* strains that clustered into that family.

**Table 1:** Overview of the number of OMPs per species and strain used in this study.

Species and strain designation	Isolated from	NCBI accession number* Chromosome	NCBI accession number* Plasmid	Number of OMPs
***Campylobacter coli***				
15-157360	human	NC_022660°	NC_022656	21
76339	human	NC_022132°		24
N29710	chicken	NC_022347°	NC_022355 and NC_02234	27
***Campylobacter jejuni***				
00-2425	human	NC_022362°		23
4031	contaminated tap water	NC_022529°		24
M1	human	NC_017280°		22
***Escherichia coli*** ^**$**^				
536	human	NC_008253°		110
IAI1	human	NC_011741°		89
IAI39	human	NC_011750°		97
K12-W3110	human	NC_007779°		88
TW14359	human	NC_013008°	NC_013010	116
***Helicobacter acinonychis***				
Hacino1	Cheetah	FZMD00000000°°		95
Hacino2	Sumatran tiger	FZLX00000000°°		92
Hacino3	Lion	NC_008229°	NC_008230	106
Hacino4	Bengal tiger	FZLV00000000°°		94
***Helicobacter ailurogastricus***				
ASB7	cat	CDMG00000000°°		59
ASB9	cat	CDMN00000000°°		60
ASB11	cat	CDML00000000°°		60
ASB13	cat	CDMH00000000°°		60
***Helicobacter baculiformis***				
M50	cat	FZMF00000000°°		62
***Helicobacter bizzozeronii***				
10	dog	FZEH00000000°°		69
14	dog	FZMK00000000°°		65
CIII	human	NC_015674°	NC_015670	68
M7	dog	FZLK00000000°°		70
***Helicobacter cetorum***				
MIT 00-7128	whale	NC_017737°	NC_017738	118
MIT 99-5656	dolphin	NC_017735°	NC_017736	101
***Helicobacter cinaedi*** ^**$$**^				
BAA_847	human	NC_020555°		29
***Helicobacter cynogastricus***				
JKM4	dog	FZMQ00000000°°		62
***Helicobacter equorum***				
eqF1	horse	FZPO00000000°°		24
***Helicobacter felis***				
CS1	cat	NC_014810°		65
CS6	cat	FZKM00000000°°		66
CS7	cat	FZKX00000000°°		67
DS1	dog	FZNI00000000°°		61
***Helicobacter heilmannii***				
ASB1	cat	CDMK00000000°°		53
ASB2	cat	CDMP00000000°°		55
ASB3	cat	CDMJ00000000°°		56
ASB6	cat	CDMM00000000°°		56
ASB14	cat	CDMI00000000°°		55
***Helicobacter hepaticus***				
ATCC 51449	mouse	NC_004917°		28
***Helicobacter mustelae***				
ATCC 12198	ferret	NC_013949°		49
***Helicobacter pylori***				
Puno120	human (Amerindian)	NC_017378°	NC_017377	69
G27	human (Europe)	NC_011333°	NC_011334	69
F30	human (East Asia)	NC_017365°	NC_017369	72
J99	human (USA)	NC_000921°		71
India7	human (India)	NC_017372°		74
SouthAfrica7	human (South Africa)	NC_022130°		69
***Helicobacter salomonis***				
M45	dog	FZLZ00000000°°		47
R1053	dog	OANQ00000000°°		48
KokIII	dog	FZMA00000000°°		49
***Helicobacter suis***				
HS2	pig	FZLI00000000°°		53
HS4	pig	FZKI00000000°°		52
HS7	pig	FZKH00000000°°		53
HS9	pig	FZLE00000000°°		53
***Helicobacter trogontum***				
R3554	rat	FZNG00000000°°		55

^$$^For the gastric and the enterohepatic *Helicobacter* species, genomes were chosen based on their availability at the moment of analysis in the NCBI database.

For *H*. *pylori*, six fully annotated genomes from strains of different regions (one strain per geographical area) were selected (Smet *et al*. 2018 DOI: 10.1038/s41396-018-0199-5).

*Campylobacter* and *E*. *coli* strains were included as reference strains.

^$^For *E*. *coli*, each strain was chosen out of a different phylogroup based on the paper of Bohlin *et al*. 2014. (https://www.ncbi.nlm.nih.gov/pmc/articles/PMC4200225/).

*NCBI accession numbers are available for the species and strains with fully annotated genomes. On the basis of these genomes, OMP extraction was started.

°Complete genome.

°°Draft genome.

The remaining 586 protein sequences were then searched against the Pfam protein database, resulting in the additional classification of 308 proteins into 31 putative OMP families with unknown biological function. These families were assigned a systematic family ID from “X1” to “X31”. The names of these 31 candidate OMP families and the predicted OMPs from the *E*. *coli*, *Campylobacter* and *Helicobacter* strains that clustered among these families, are shown in Supplementary Table S2.

**Figure 1:**
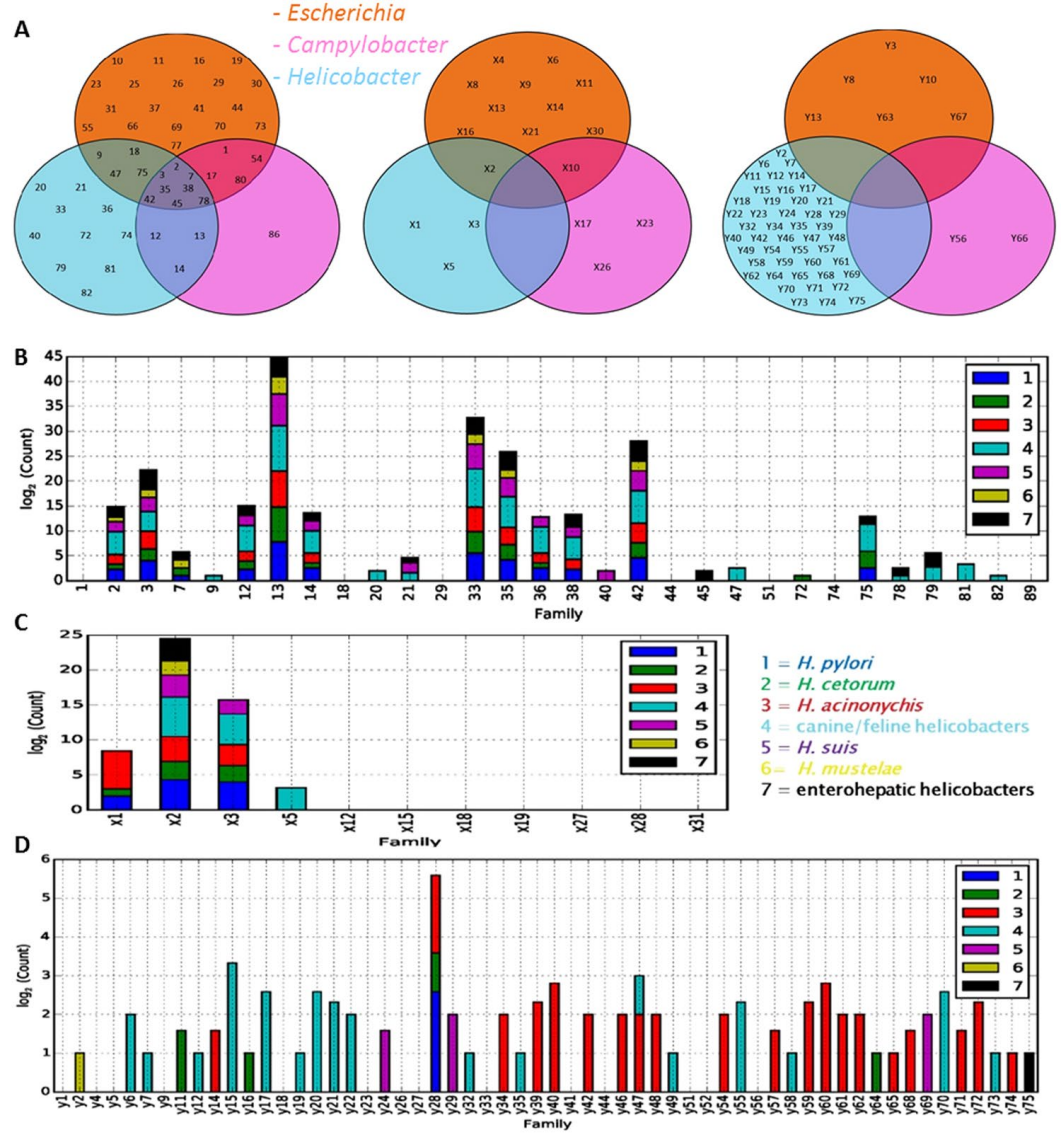
(**A**) Venn diagrams of the 90 OMPdb families, the 31 Pfam-derived “X” families and the 75 “Y” families derived from CD-HIT-generated clusters. Shown are the OMP families for the *Escherichia*, *Campylobacter-* and *Helicobacter* genera. Distribution plots of the *Helicobacter* OMPs from (**B**) the OMPdb-, (**C**) the “X”- and (**D**) the “Y” families. Families consisting of at least one OMP were included in the plots. Dark blue = *H*. *pylori*, green = *H*. *cetorum*, red = *H*. *acinonychis*, light blue = canine and feline gastric helicobacters, purple = *H*. *suis*, yellow = *H*. *mustelae*, black = enterohepatic helicobacters. For these 7 groups, the numbers of members (log2 values) in each OMP family are shown. For families with only one OMP member, bars are invisible on the log_2_ scale.

Finally, the remaining 278 unclassified protein sequences were clustered into phylogenetic groups using the CD-HIT program with algorithm settings to produce the fewest possible number of clusters. With this method, 106 clusters were produced. The clusters with single members that had a length of less than 120 amino acids (a total of 52 sequences) were excluded. This final clustering revealed 75 families of unknown annotation or function, which were assigned a systematic family ID of “Y1” to “Y75” (Supplementary Table S3). To visualize the relative distribution of the OMP families amongst *E*. *coli* and the *Campylobacter* and *Helicobacter* species, the predicted OMPdb families, and X and Y families were presented in Venn diagrams (Fig. 1A). The OMP families from the OMPdb database seemed to be well-conserved among *E*. *coli* and *Helicobacter*, and to a lesser extent in *Campylobacter* (Fig. 1A). From the 90 OMPdb families, 19 families were unique for *E*. *coli*, 10 families for the *Helicobacter* species, and only 1 family for the *Campylobacter* species. Several of the X-families from the Pfam database were found to be genus-specific (Fig. 1A). From the 31 X-families, 10 families were unique for *E*. *coli*, 3 families for *Campylobacter*, and 3 for *Helicobacter*. From the 75 Y-families, 46 families were unique for *Helicobacter* (especially in the helicobacters from cats and dogs), 2 families for *Campylobacter* and 6 were unique for *E*. *coli* (Fig. 1A).

Furthermore, distribution plots of OMPs were made to search for distinguishing components of the OMP proteomes in gastric canine/feline helicobacters, *H*. *suis*, *H*. *pylori*, *H*. *cetorum*, *H*. *acinonychis*, *H*. *mustelae*, and enterohepatic helicobacters (Fig. 1B–D). Nine families of the OMPdb database were found well-conserved among gastric and enterohepatic helicobacters (Family 2,3,12,13,14,33,35,38 and 42), and one family (Family 36) was conserved among the gastric species only. The other 20 families were specific to only a few species and strains (Fig. 1B, Supplementary Table S1). From the X-families, only family X2 was well-conserved among the genus *Helicobacter* and family X3 among the gastric species. OMPs from family X1 were only found in *H*. *pylori*, *H*. *cetorum*, and *H*. *acinonychis* and OMPs from family X5 in *H*. *felis* and *H*. *bizzozeronii*. OMPs from the other 7×-families were only present in one *Helicobacter* species (Fig. 1C, Supplementary Table S2). Each Y-family was mainly composed of OMPs from only one *Helicobacter* species. Most of these Y-families were found in canine and feline gastric helicobacters and in *H*. *acinonychis* (Fig. 1D, Supplementary Table S3). In following paragraphs, we elaborate on OMP families of particular interest for *Helicobacter* infections and disease.

### *Helicobacter*-specific OMP families with known or putative roles in virulence or colonization

#### Family 13 - *Helicobacter* Outer Membrane Protein Family 1 (Hop/Hor/Hom)

**Figure 2:**
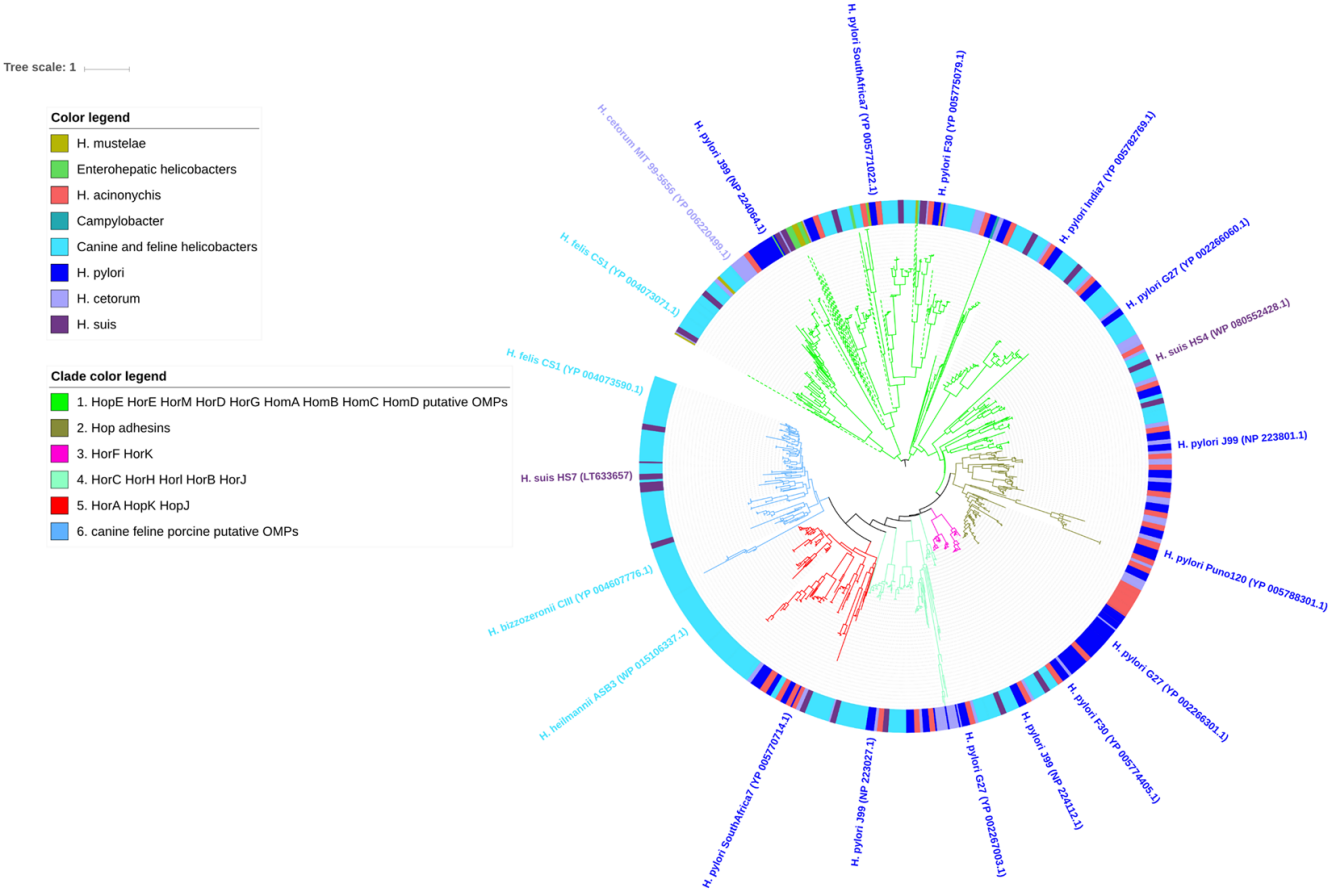
Phylogenetic tree of Family 13 – the *Helicobacter* outer membrane protein family 1. The *H*. *pylori* Hop, Hor and Hom adhesins, including BabA (HopS), SabA (HopP), AlpA (HopC) and AlpB (HopB), OipA (HopH), HopZ, HopQ, LabA (HopD), HorB and HomB clustered into this family. Orthologous OMPs are present in all examined gastric and enterohepatic *Helicobacter* species, only one orthologous protein is present in *C*. *coli* (76339) and *C*. *jejuni* (00-2425) and there are no orthologous OMPs detected in *E*. *coli*. Indicated are 6 different subgroups: putative OMPs specific for canine, feline and porcine helicobacters (6); *H*. *pylori* HorA, HopK and HopJ (5) with orthologous putative OMPs in all other gastric NHPH species, except *H*. *mustelae*; *H*. *pylori* HorC, HorH, HorI, HorB and HorJ (4) with orthologous putative OMPs in all other gastric NHPH species, except *H*. *mustelae*; *H*. *pylori* HorF and HorK (3) with orthologous putative OMPs in other gastric NHPH species, except *H*. *baculiformis* and *H*. *mustelae*; *H*. *pylori* Hop adhesins (2) that are absent in other gastric NHPH species except for *H*. *acinonychis* and *H*. *cetorum*; *H*. *pylori* HopE, HorE, HorM, HorD, HorG, HomA, HomB, HomC and HomD (1) with orthologous putative OMPs in all other gastric NHPH species. Also, the 1 orthologous OMP from *C*. *coli* (76339) and *C*. *jejuni* (00-2425) (dark cyan) cluster in the latter subgroup, as well as a few OMPs from *H*. *mustelae* (yellow-green) and enterohepatic helicobacters (green). The position of the different *H*. *pylori* OMPs in each subgroup are clockwise indicated. OMPs of *H*. *mustelae*, enterohepatic helicobacters and *Campylobacter* species are indicated by dashed clade lines. Per clade, minimum one accession number is added.

A total of 1,155 orthologous OMPs were clustered among the *Helicobacter* outer membrane protein family 1 (Family 13 of the OMPdb database, Supplementary Table S1). This family encompasses the *H*. *pylori* Hop, Hor and Hom proteins. Orthologs were present in all examined gastric and enterohepatic *Helicobacter* species. Only one orthologous protein was present in *C*. *coli* (76339) and *C*. *jejuni* (00–2425) and there were no members of this family detected in *E*. *coli*. The phylogenetic tree of this OMP family shows 6 subgroups (clades) could be distinguished (Fig. 2). Strikingly, the known Hop adhesins (2) all cluster into a monophyletic clade (Fig. 2, clade 2) that is specific for *H*. *pylori* (dark blue), its closest relative *H*. *acinonychis* (red), and *H*. *cetorum* (light purple) and which were found to be completely absent in canine, feline or porcine gastric NHPH. In *H*. *pylori* family 13, clade 2 comprises several group members, of which AlpA, AlpB, HopA, HopF, HopI, HopG, HopL and OipA were found to be conserved in *H*. *acinonychis* and *H*. *cetorum*, which additionally held orthologs of BabB and HopD, and of SabA respectively (Fig. 2). BLAST analyses revealed that several putative OMPs from the canine, feline and porcine gastric NHPH species clustered into these subgroups. For the enterohepatic *Helicobacter* species and *H*. *mustelae*, only orthologous putative OMPs of HorD and HorG were present in this family (clade 1 in Fig. 2). The orthologous OMP from *C*. *coli* (76339) and *C*. *jejuni* (00-2425) also clustered in this latter subgroup. Interestingly, subgroup 6 (Fig. 2) contained putative OMPs that were present in canine, feline and porcine *Helicobacter* species only.

#### Family 33 - *Helicobacter* Outer Membrane Protein Family 2 (Hof)

**Figure 3:**
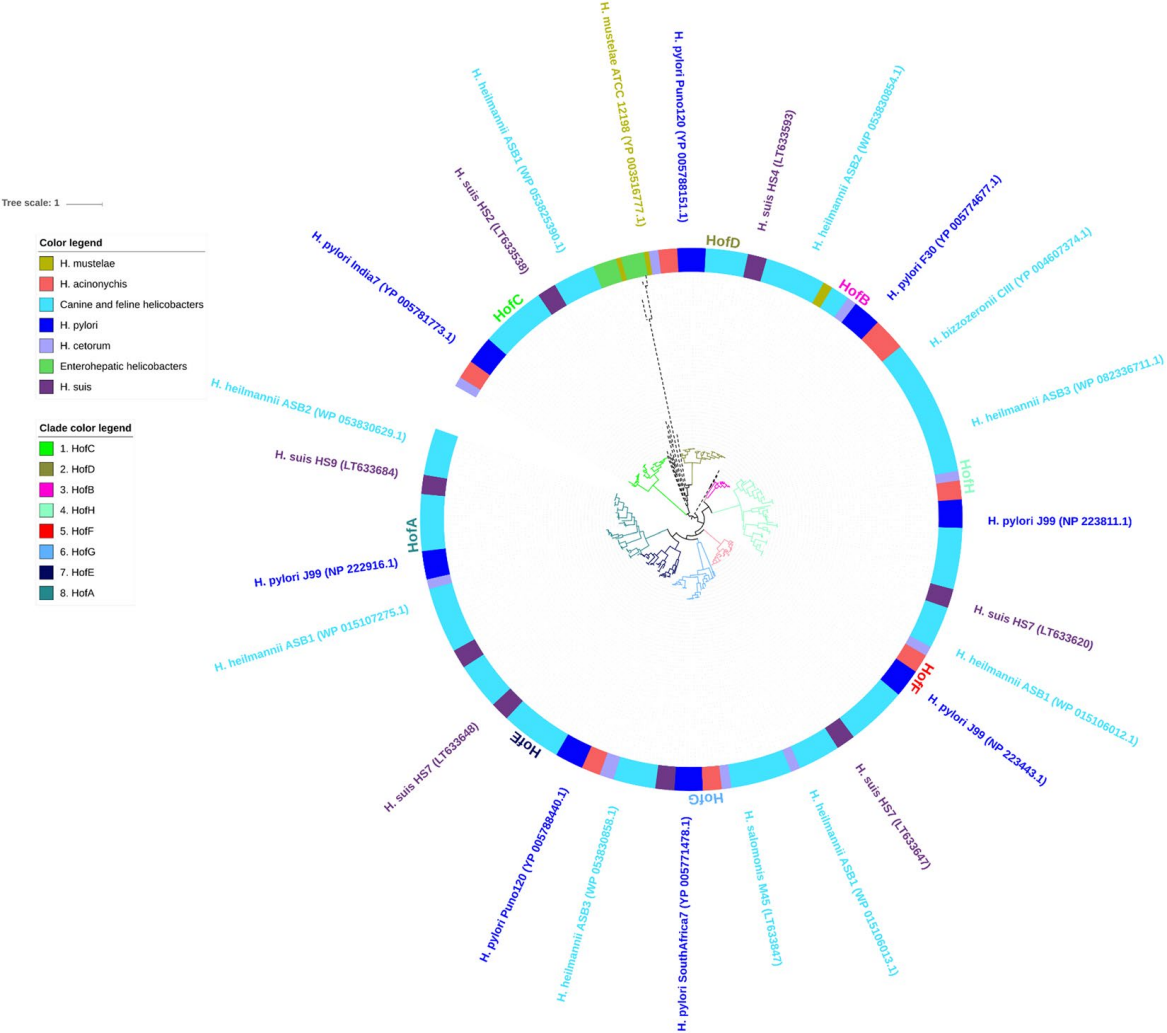
Phylogenetic tree of Family 33 – the Helicobacter outer membrane protein family 2. Orthologous OMPs are present in all examined *Helicobacter* species, but are absent in *Campylobacter* and *E*. *coli*. Indicated are 8 different subgroups, corresponding to the different *H*. *pylori* Hof proteins: HofA (8) with orthologous putative OMPs in gastric NHPH except *H*. *acinonychis*, *H*. *salomonis* and *H*. *mustelae*; HofB (3) with orthologous putative OMPs in *H*. *acinonychis*, *H*. *bizzozeronii* and *H*. *cetorum*; HofC (1), HofD (2), HofE (7), HofF (5), HofG (6) and HofH (4) with orthologous putative OMPs in all gastric *Helicobacter* species except *H*. *mustelae*. *H*. *mustelae* (yellow-green) and enterohepatic helicobacters (green) contain a few uncharacterized OMPs that cluster separately. OMPs of *H*. *mustelae* and enterohepatic helicobacters are indicated by dashed clade lines. Per clade, minimum one accession number is added.

A total of 347 orthologous OMPs clustered in the *Helicobacter* outer membrane protein family 2 (Family 33 of the OMPdb database, Supplementary Table S1). This family comprises orthologs of the *H*. *pylori* Hof proteins. Members of these Hof OMPs were present in all examined *Helicobacter* species and absent in *C*. *coli*, *C*. *jejuni* or *E*. *coli*. The phylogenetic tree of this OMP family reveals 8 subgroups, corresponding with the *H*. *pylori* HofA, HofB, HofC, HofD, HofE, HofF, HofG and HofH proteins (Fig. 3A). These 8 subgroups were highly conserved among *H*. *acinonychis*, *H*. *cetorum*, as well as canine, feline and porcine NHPH species, except for HofB, which is only present in *H*. *bizzozeronii*. On the contrary, the analysed enterohepatic helicobacters and *H*. *mustelae* only contained a few putative Hof-like OMPs, which did not cluster with one of the 8 Hof proteins known from *H*. *pylori*.

Hof proteins lack close homologs outside the genus *Helicobacter*. They are of unknown function except for reports in *H*. *heilmannii* that implicate HofE and HofF in adherence to human gastric mucins^19^. To gain insight into the putative Hof function, representative sequences of the 8 Hof subgroups were subjected to remote homology and fold recognition searches using the Protein Homology/analogy Recognition Engine 2 (PHYRE2)^24^ and RaptorX^25^. Both algorithms picked up high confidence structural homology to 18-stranded β-barrels of the Outer membrane Carboxylate Channel (Occ) family proteins (formerly Opd/Opr) found in *Pseudomonas* and *Acinetobacter*^26,27^, as well as to the 18-stranded Major Outer Membrane Protein (MOMP) of *C*. *jejuni*^28^. In support of this homology assignment, the transmembrane elements in the 3D threaded structure are also found by the transmembrane β-barrel protein predictor BOCTOPUS^29^, have plausible hydrophobic residue distributions, and show the presence of 2 aromatic “belts” as is frequently observed in OM β-barrels (Supplementary Fig. S1). The Occ family OMPs are monomeric 18-stranded porins found in species lacking large channel trimeric porins, and are implicated in non-specific diffusion of small carboxylate-containing solutes across the OM^26,27^. *C*. *jejuni* MOMP can be found both as a monomeric or trimeric porin of 18-stranded β-barrels involved in cation selective passive diffusion over the OM^30^, though has also been implicated in bacterial adherence^31^.

#### Family 36 - Systemic factor protein A (SfpA/LpxR)

The prototype of this family is the systemic factor protein A (SfpA) of *Yersinia enterocolitica*. Lipid A deacylase (LpxR), a SfpA homologue in *Salmonella* Typhimurium, has been shown to be important for immune evasion through lipopolysaccharide modification^32^. Also in *H*. *pylori*, LpxR homologs play a key role in the establishment of long-term colonization^33^.

**Figure 4:**
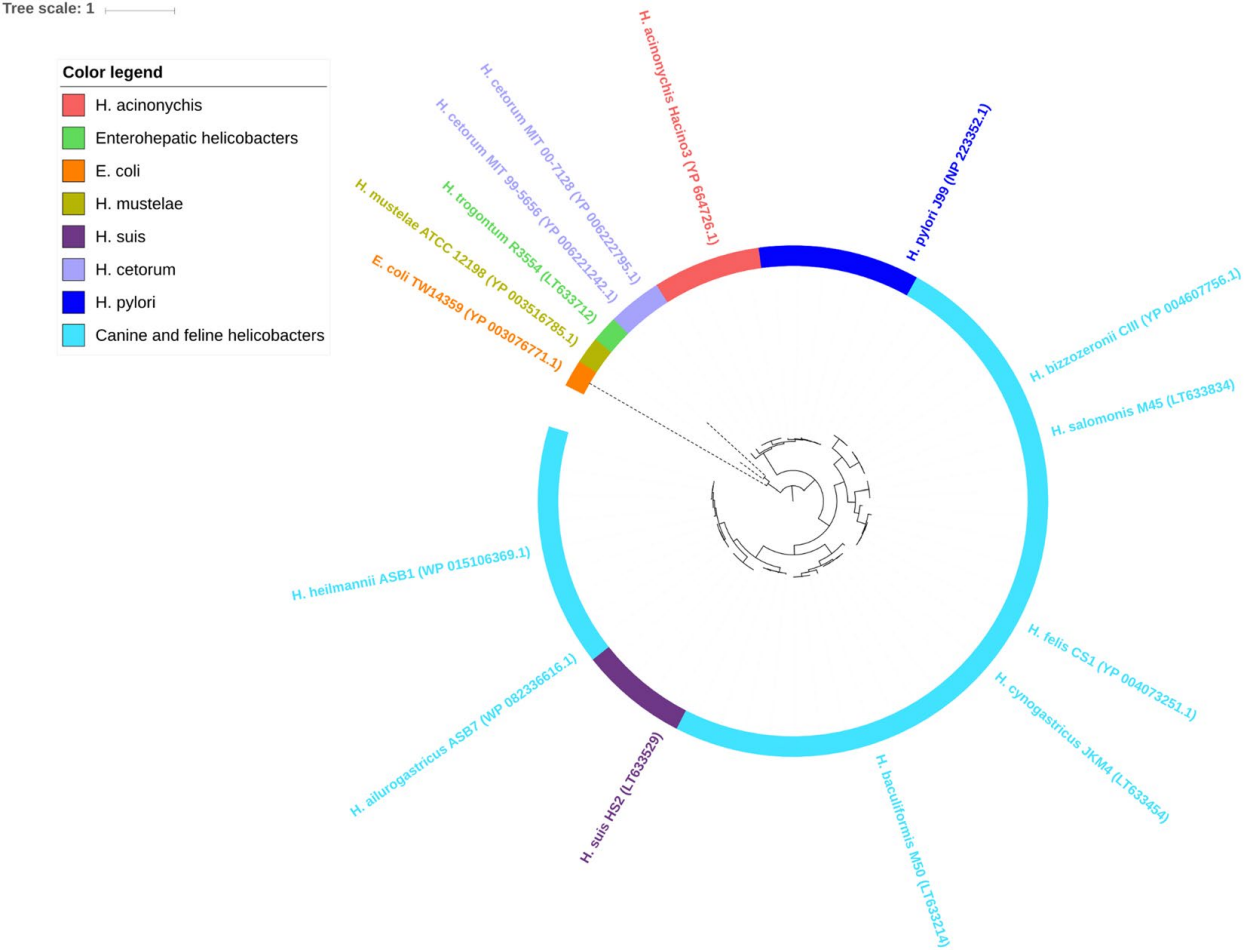
Phylogenetic tree of Family 36 – Systemic factor protein A (SfpA/LpxR). Orthologous OMPs are present in the examined strains of *Helicobacter* except for *H*. *cinaedi*, *H*. *equorum* and *H*. *hepaticus*, and only one orthologous OMP is present in *E*. *coli* strain TW14359 (orange). In the other *E*. *coli* strains and in *Campylobacter*, no orthologous OMPs are detected. *H*. *mustelae* (yellow-green) clusters closer to the enterohepatic species *H*. *trogontum* (green) than to gastric NHPH species. OMPs of *H*. *mustelae* and enterohepatic helicobacters are indicated by dashed clade lines. Per clade, minimum one accession number is added.

Except for *H*. *cinaedi*, *H*. *equorum*, and *H*. *hepaticus*, the examined *Helicobacter* strains contained between 1 and 2 orthologs of SfpA/LpxR per genome (Family 36 of the OMPdb database, Supplementary Table S1), suggesting that LPS remodeling by lipid A deacylation is a common characteristic in most *Helicobacter* species. In contrast, only one orthologous protein was present in *E*. *coli* strain TW14359 and there were no members of the SfpA/LpxR family detected in *Campylobacter* and the other *E*. *coli* strains (Supplementary Table S1). As shown in the phylogenetic tree (Fig. 4), the *H*. *mustelae* ortologous OMP clustered closer to enterohepatic helicobacters than to gastric NHPH species.

#### Family X1 and X3 – (Putative) vacuolating cytotoxin family

The secreted vacuolating cytotoxin A (VacA), belonging to the autotransporter OMP family, is an important virulence factor of *H*. *pylori*. After binding to and internalization into host epithelial cells, VacA induces cellular vacuolation and various other responses^34,35^. Strikingly, most other *Helicobacter* species lack VacA homologs, except for *H*. *cetorum* and *H*. *acinonychis*.

**Figure 5:**
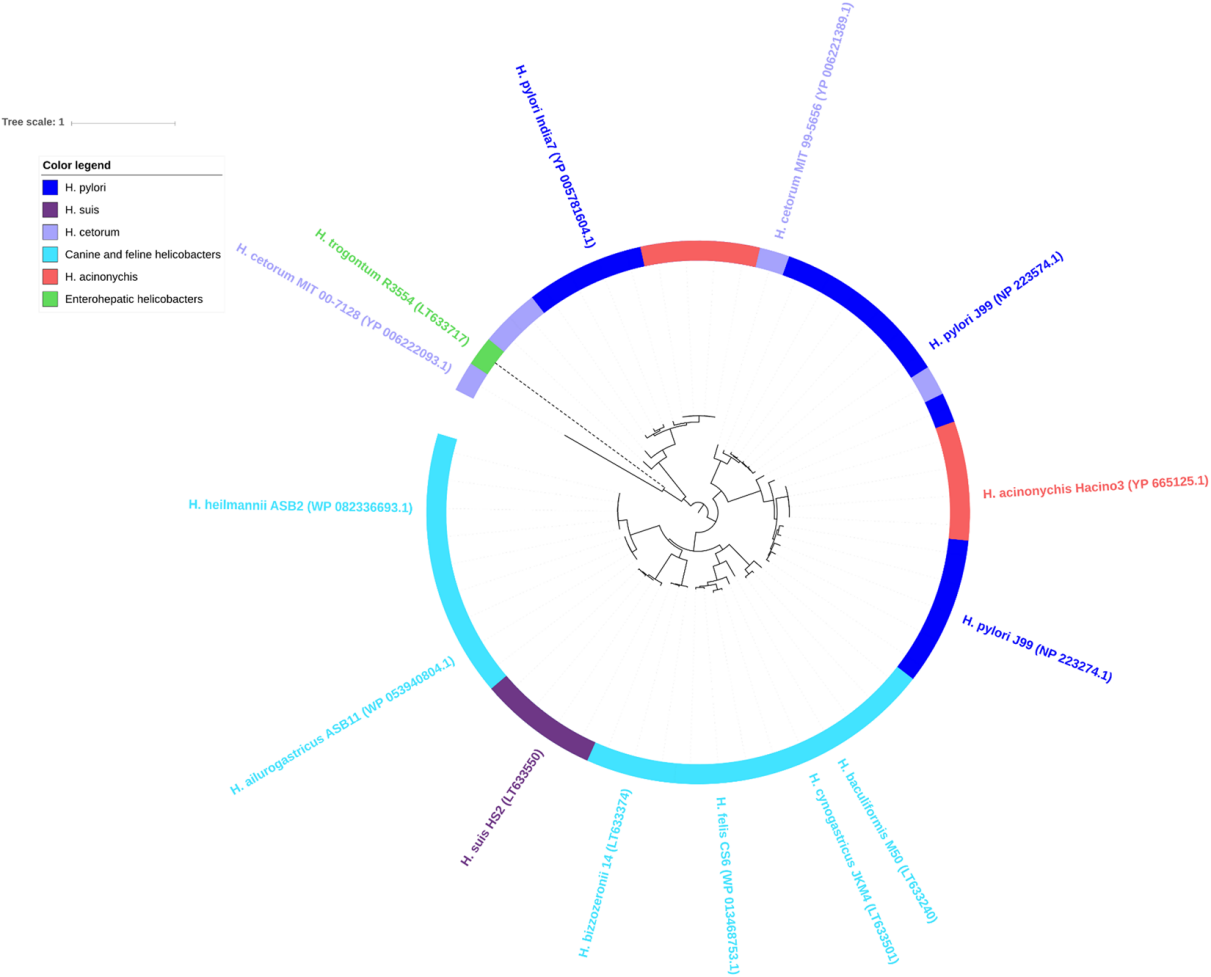
Phylogenetic tree of Family X3 – putative vacuolating cytotoxin. Orthologous OMPs are present in the examined strains of *Helicobacter* except for *H*. *cinaedi*, *H*. *equorum*, *H*. *hepaticus*, *H*. *mustelae* and *H*. *bizzozeronii* 10. Orthologous OMPs are absent in *E*. *coli* and *Campylobacter* species. Most *Helicobacter* strains harbor one VacA-like protein, whereas *H*. *acinonychis*, *H*. *cetorum* MIT 00-7128 and *H*. *pylori* Puno120 and F30 harbor two copies, and *H*. *cetorum* MIT 99-5656 and *H*. *pylori* G27, J99, India7 and SouthAfrica harbor tree copies of the VacA-like OMP. OMPs of enterohepatic helicobacters are indicated by dashed clade lines. Per clade, minimum one accession number is added.

In the present study, all analysed *H*. *pylori* and *H*. *cetorum* strains each harbored one *vacA* copy (Family X1 of the Pfam database, Supplementary Table S2), whilst *H*. *acinonychis* contained more than one *vacA* copy, though these were inactivated by insertion sequences compared to *H*. *pylori* and *H*. *cetorum* as described before^15,36^. Besides VacA, *H*. *pylori* also contains 3 VacA-like autotransporters that each enhance its capacity to colonize the stomach^35^. Previously, we reported the presence of such *vacA*-like autotransporter gene in canine, feline and porcine gastric *Helicobacter* species^37^. At the protein level, these VacA-like autotransporters showed to be highly divergent amongst the different *Helicobacter* species^15^. In the present study, 55 homologs of the VacA-like protein were classified into the putative vacuolating cytotoxin family (Family X3 of the Pfam database, Supplementary Table S2). Orthologs were present in the examined strains of *Helicobacter* except for *H*. *cinaedi*, *H*. *equorum*, *H*. *hepaticus*, *H*. *mustelae* and *H*. *bizzozeronii* 10. There were no orthologs of this autotransporter present in *Campylobacter* and *E*. *coli* (Fig. 5).

### Well-conserved OMP families present among Helicobacter, Campylobacter and/or *E. coli* with a role in virulence or colonization

#### Family 14 - Imp/OstA

Imp (increased membrane permeability) or OstA (organic solvent tolerance) is an organic solvent tolerance protein in Gram-negative bacteria that participates in outer membrane biogenesis and integrity^38,39^. The Imp/OstA protein is implicated in the translocation and insertion of LPS into the outer leaflet of the OM bilayer. It has also been associated with membrane permeability, organic solvent tolerance and resistance to antibiotics in *H*. *pylori*^40,41^. We identified 48 orthologous outer membrane proteins in the Imp/OstA family (Family 14 of the OMPdb database, Supplementary Table S1). In each of the examined strains of *Campylobacter* and *Helicobacter*, except for *H*. *trogontum*, one orthologous Imp/OstA protein was found. However, the latter species harbored an additional putative OstA paralog (Family X18, Supplementary Table S2). The phylogenetic tree of OMP family 14 is shown in Supplementary Fig. S2. In accordance to the other families, *H*. *mustelae* clustered closer to enterohepatic helicobacters than to gastric NHPH species.

#### Family 42 - Outer membrane factor (OMF)

Gram-negative bacteria possess energy-dependent transport systems to export proteins, carbohydrates, drugs and heavy metals across the two membranes of the cell envelope^42^. Type 1 protein secretion systems (e.g. HlyBD-TolC) and RND multidrug efflux systems (e.g. AcrA/B-TolC or MexAB-OprM) consist of a cytoplasmic or inner membrane (IM) export system, a membrane fusion protein (MFP) and an outer membrane factor (OMF). TolC, the prototype OMF found in *E*. *coli* forms a trimeric 12-stranded β-barrel (3 × 4 strands) with an extended coiled coil domain reaching into the periplasm and contacting the IM-localized export system via the MFP^43^. These transport systems have been shown to play a role in protein export and multidrug efflux, the latter producing both intrinsic and elevated multidrug resistance^42,44,45^. In total, 219 orthologous proteins were identified to belong to the OMF family (Family 42 of the OMPdb database, Supplementary Table S1). Different members of the OMF family were present in all the analysed *E*. *coli*, *Campylobacter* and *Helicobacter* strains. With protein BLAST, several different OMPs subgroups could be distinguished in the OMF family (Supplementary Table S4). These different subgroups are indicated in the phylogenetic tree of the OMF family (Supplementary Fig. S3). Also, here, orthologs of the OMP family of *H*. *mustelae* clustered with enterohepatic helicobacters rather than with gastric *Helicobacter* species.

#### Family 3 - Outer Membrane Receptor (OMR-TonB Dependent Receptor) and Family X2 - TonB-dependent Receptor Plug Domain

TonB-dependent transporters are bacterial outer membrane proteins that bind and transport ferric chelates called siderophores, as well as vitamin B_12_, nickel complexes, and carbohydrates into the periplasm. For which they use energy from the proton motive force of the cytoplasmic membrane via the TonB-ExhB-ExbD membrane proteins. The TonB-dependent outer membrane receptors have also been shown to be required for bacterial virulence^46^. In the present study, two TonB-dependent outer membrane receptor families were identified.

Family 3 of the OMPdb database comprised the TonB-dependent outer membrane receptors, including Btub, CfrA, FhuA, FhuE, Fiu, FecA, FepA, and FerA, for the uptake of iron (siderophores), nickel and vitamin B_12_. A total of 123 orthologous OMP proteins clustered in this family. Members of this family are present in all examined strains of *E*. *coli*, *Campylobacter* and *Helicobacter* except for *H*. *bizzozeronii* and *H*. *salomonis*. *Helicobacter* genomes contained an average of 2 paralogs per strain. The phylogenetic tree of OMP family 3 is shown in Supplementary Fig. S4. The TonB-dependent receptors of *H*. *mustelae* clustered together with those of enterohepatic helicobacters which on their turn clustered together with the *Campylobacter* TonB-dependent receptors.

Family X2 of TonB-dependent receptors comprised 116 orthologous proteins (Family X2 of the Pfam database, Supplementary Table S2). Members of this family were present in all examined strains of *E*. *coli* and *Helicobacter* apart from *H*. *salomonis*. OMPs belonging to this TonB-dependent receptor family were absent in *C*. *coli* and *C*. *jejuni*. The phylogenetic tree of OMP family X2 is shown in Supplementary Fig. S5.

#### Family 38 - Outer Membrane Phospholipase (OMPLA)

The outer membrane phospholipase A (OMPLA), encoded by the *pldA* gene which is widespread among Gram-negative bacteria, hydrolyses acyl ester bonds in phospholipids and lysophospholipids^47,48^. OMPLA has been described as a virulence factor. For instance, in *C*. *coli*, OMPLA was identified as a major hemolytic factor and *H*. *pylori* OMPLA has been shown to be involved in the colonization and invasion of the human gastric mucosa^47,49–52^. Moreover, *H*. *pylori* isolates with high OMPLA activity have been associated with peptic ulcer disease in human patients^53,54^. In the present study, 53 proteins were classified in the OMPLA family (Family 38 of the OMPdb database, Supplementary Table S1). All *E*. *coli*, *Campylobacter* and *Helicobacter* strains, apart from *H*. *mustelae*, *H*. *equorum*, *H*. *cetorum* MIT 00-7128, and *H*. *pylori* G27, harbored 1 OMPLA ortholog, whereas 4 different paralogs were present in *H*. *trogontum*. Phylogenetically, the OMPLA orthologs of all strains clustered separately per genera and per species (Supplementary Fig. S6).

## Discussion

In this study, we have analysed the genome sequences from a total of 54 different strains of the genera *Helicobacter*, *Campylobacter* and *E*. *coli* for their presence of OMPs. Considering the genome length and the total protein count, gastric *Helicobacter* species harbor proportionally more OMPs than other helicobacters or the *Campylobacter* or *Escherichia* reference genomes. Gastric helicobacters have small genomes and proteomes compared to the average *E*. *coli* genome, which holds a median genome length and protein count of, respectively, 5.17 Mb and 4931 proteins according to the NCBI database. Instead, *H*. *pylori* has a median genome length of only 1.63 Mb with a median protein count of 1451. However, *H*. *pylori*’s surface-localized proteome did not shrink proportionally, and ~4% of its total protein count constitutes of OMPs, compared to just ~2% for *E*. *coli*. This large collection of OMPs in an otherwise reductionist genome suggests they form important fitness factors in the survival and adaptation to the harsh gastric environment^4^. Among the gastric *Helicobacter* species, the total OMP number was the highest for the *H*. *acinonychis* (a mean of 97) and *H*. *cetorum* strains (a mean of 110), although it should be noted that these species contain multiple fragmented OMPs (Table 1).

In general, we found that the clustering of a strain or species’ OMPs in the phylogenetic trees is similar to the phylogenetic clustering of their full genomes. The phylogenetic reconstructions of the different families revealed a clear and evident division between enterohepatic and gastric *Helicobacter* species. The gastric helicobacters could be further divided into *H*. *pylori* and its two closest relatives, *H*. *acinonychis* and *H*. *cetorum* and NHPH species including the canine, feline, and porcine helicobacters clades. Interestingly, *H*. *mustelae*, which has been associated with gastritis, peptic ulcers, MALT lymphoma, and adenocarcinoma in domestic ferrets^55,56^, clustered within the clade of the enterohepatic *Helicobacter* species. This supports previous hypotheses, which emphasize the capability of *H*. *mustelae* to colonize both the stomach and the intestinal tract^15^.

The primary function of the outer membrane of Gram-negative bacteria is to form a barrier against hazardous substances from the environment such as enzymes, detergents, and antimicrobials. The permeability of the outer membrane is determined by the presence of OMPs that function as porins. They contain transmembrane diffusion channels through which small hydrophilic molecules, nutrients, and small antibiotics can be transported across the outer membrane^4,57^. In our study, two *Helicobacter*-specific porin families were found, namely Family 13 and 33 (Supplementary Table S1, Figs 2 and 3). Both families mainly contain OMP orthologs from the genus *Helicobacter* albeit with a greater extent in the gastric helicobacters than in the enterohepatic ones. *C*. *coli* and *C*. *jejuni* only harbor one such OMP and members of these families are even lacking in *E*. *coli*. The families 13 and 33 were thus probably acquired by *Helicobacter* after splitting-off from a last common ancestor. Moreover, most gastric NHPH species lack all *H*. *pylori* Hop adhesins, suggesting that these OMPs were acquired after *H*. *pylori* speciation^15–18^. The *H*. *pylori*-specific Hop proteins function as adhesins for gastric epithelial cells^4^. Interestingly, the adhesive properties of the blood group antigen binding adhesin BabA was found to be pH responsive and to provide the bacteria with a reversible adherence profile that is fine-tuned to the pH-gradients in the stomach mucosa^58^. Also the canine, feline and porcine gastric NHPH species have been shown to attach to the gastric mucosa^15,19^. The absence of the *H*. *pylori* Hop adhesins in these NHPHs suggests that other OMPs function as adhesins in these organisms. Indeed, genes encoding orthologs of *H*. *pylori* Hof proteins seem to be well conserved in the canine, feline and porcine gastric NHPH species of which HofE and HofF have recently been identified as adhesins in *H*. *heilmannii*^19^. HofF has also been shown to be important for *H*. *pylori* colonization, but the function of the other *H*. *pylori* Hof OMPs remains largely unknown^19,59^. Furthermore, the exact role of the other NHPH OMPs from Families 13 and 33 in NHPH colonization remains to be further elucidated. Remote homology recognition and 3D threading of family 33 (Hof) members indicates structural similarity with 18-stranded porins of the Occ family in *Pseudomonas* and *Acinetobacter*, and the *C*. *jejuni* major outer membrane protein – MOMP, which are implicated in cation-selective solute diffusion across the OM, as well as in adherence in case of MOMP^28,30,31^.

The explicit OMP variation among species might also be favorable for evasion of the host’s immune response. *H*. *pylori* has developed a very large repertoire of mechanisms to evade both innate and adaptive immune recognition^60^. One way of *H*. *pylori* to evade the immune response is the avoidance of recognition by Toll-like receptors of its bacterial surface molecules such as LPS and flagellin. Here, we identified the SfpA/LpxR OMP family (Family 36, Supplementary Table S1, Fig. 4) present in all gastric *Helicobacter* species, which might be involved in immune evasion by removing the 3′-acyloxyacyl group of lipid A^61,62^. For the enterohepatic *Helicobacter* species tested here, SfpA/LpxR was only detected in *H*. *trogontum*. This OMP family might thus be more specific for gastric species than their enterohepatic members within the genus *Helicobacter*.

A very well-studied outer membrane virulence factor of *H*. *pylori* is the secreted vacuolating cytotoxin A (VacA; Family X1, Supplementary Table S2) that causes uncontrolled cellular vacuolation^34,35^. In agreement with previous research^15,36^, we identified the VacA OMP only in *H*. *pylori* and *H*. *cetorum* and short fragments of this protein in *H*. *acinonychis*, but not in other NHPH species. We additionally found a *Helicobacter*-specific putative VacA-like cytotoxin family (Family X3, Supplementary Table S2, Fig. 5). The VacA-like autotransporters belonging to this family enhance the bacterium’s colonization capacity of the stomach^63^. The VacA-like OMP is well conserved among the different gastric *Helicobacter* species, although their protein sequences exhibit much variation^15^. Besides, we also showed variation in the number of VacA-like autotransporters, not only between species but also at the species level. For instance, in *H*. *bizzozeronii* strain 10, no VacA-like autotransporter could be identified, whereas the other examined strains of *H*. *bizzozeronii* each contained one copy of this OMP. This underlines the genetic diversity among strains within a species. However, it should be noted that the genomes of most NHPH species that were analysed in this study, including that of *H*. *bizzozeronii* strain 10, are draft genomes that lack approximately 5% of the full genome sequence. Therefore, it cannot be excluded that the gene encoding the VacA-like autotransporter of this *H*. *bizzozeronii* strain is part of the lacking 5% of its genome sequence. The genomes of enterohepatic *Helicobacter* species, except for *H*. *trogontum*, lack a VacA-like autotransporter as well. This OMP family may therefore be more specific for gastric helicobacters. However, in the *H*. *mustelae* strain included in our study, the VacA-like OMP is absent as well. Also for all other families that were analysed, the OMPs of this *H*. *mustelae* strain clustered closer together with enterohepatic helicobacters, or separately between enterohepatic and gastric species. Thus, although *H*. *mustelae* has been described as a gastric *Helicobacter* species associated with gastric malignancies, recent studies suggest that this species is an enterohepatic species by origin, but adapted its colonization niche from the intestinal to the gastric environment^23,56,64,65^.

In addition, several OMP families were found to be well conserved among *E*. *coli* and the *Campylobacter*, and *Helicobacter* genera. The Imp/OstA family (Family 14, Supplementary Table S1, Fig. S2), an organic solvent tolerance protein, and OMF family (Family 42, Supplementary Table S1, Fig. S3), part of type 1 protein secretion systems and RND multidrug efflux systems, maintain a barrier for antimicrobial agents and play a role in the resistance to drugs. In contrast to the *Helicobacter*-specific outer membrane porins which utilize passive diffusion for solute uptake, outer membrane receptor proteins, such as TonB-dependent receptors, carry out high-affinity binding and energy-dependent uptake of specific substrates including iron. In this study, two TonB-dependent receptor families (Family 3 and Family X2, Supplementary Tables S1 and S2, Figs S4 and S5) were identified that contribute to bacterial virulence^46^. Remarkably, OMPs from both Family 3 and Family X2 were lacking in *H*. *salomonis*. This may suggest that this species has other iron uptake mechanisms at their disposal to maintain iron homeostasis.

Finally, a virulence factor that has been shown to influence the colonization capacity and pathogenicity of Gram-negative bacteria, is the outer membrane phospholipase A (OMPLA) (Family 38, Supplementary Table S1, Fig. S6). Orthologs of this family could not be found in the genomes of *H*. *cetorum* strain MIT 00-7128, *H*. *pylori* strain G27, *H*. *equorum*, and *H*. *mustelae*. Whether the absence of OMPLA in these strains influences their virulence and colonization capacity remains to be further investigated.

In conclusion, several important OMP families, mainly from gastric *Helicobacter* species, were determined by using comparative genomic and phylogenetic analyses. To our knowledge, this is the first report analysing OMP occurrence and diversity in NHPH species, since previous studies on the OMP repertoire in the genus *Helicobacter* have mostly concentrated on the human pathogen *H*. *pylori*. Two *Helicobacter*-specific outer membrane protein families with possible functions in adhesion (Family 13, i.e. *Hop*, *Hor* and *Hom*; and Family 33, *Hof*), the *Helicobacter-*specific SfpA/LpxR OMP (Family 36) that functions in immune evasion, and a *Helicobacter*-specific VacA-like cytotoxin family with a role in colonization capacity (Family X3), were identified primarily in gastric species. Furthermore, we showed that most *Helicobacter* species contain an outer membrane factor (OMF; Family 42) and Imp/OstA (Family 14), both involved in antimicrobial resistance, TonB-dependent OMPs with a function in metal and vitamin-uptake (Family 3 and Family X2), and an outer membrane phospholipase (OMPLA; Family 38) that plays a role in colonization capacity.

In summary, our systemic survey of *Helicobacter* OMPs points to species and infection-site specific members that are interesting candidates for future virulence and colonization studies.

## Methods

### *Escherichia coli*, Campylobacter and *Helicobacter* species included

For the gastric and the enterohepatic *Helicobacter* species, genomes were chosen based on their availability at the moment of analysis. Therefore, the genomes from 12 gastric *Helicobacter* species, namely *H*. *acinonychis* (4 strains), *H*. *ailurogastricus* (4 strains), *H*. *baculiformis* (1 strain), *H*. *bizzozeronii* (4 strains), *H*. *cetorum* (2 strains), *H*. *cynogastricus* (1 strain), *H*. *felis* (4 strains), *H*. *heilmannii* (5 strains), *H*. *mustelae* (1 strain), *H*. *pylori* (6 strains), *H*. *salomonis* (3 strains), and *H*. *suis* (4 strains) and from 4 enterohepatic *Helicobacter* species, namely *H*. *cinaedi* (1 strain), *H*. *equorum* (1 strain), *H*. *hepaticus* (1 strain), and *H*. *trogontum* (1 strain) were analysed in this study. Since several genomes are described for *H*. pylori, we selected six fully annotated ones from strains of different geographical regions as described by Smet *et al*. (2018, DOI: 10.1038/s41396-018-0199-5). For comparison, the genomes from the *Campylobacter* species *C*. *coli* (3 strains) and *C*. *jejuni* (3 strains), which are closely related to *Helicobacter*, were included as well. Also, the genomes from different *E*. *coli* strains (5 strains) were analysed, since the biological functions of most *E*. *coli* OMPs are well known. Each strain was chosen out of a different phylogroup based on the paper of Bohlin *et al*. (2014, https://www.ncbi.nlm.nih.gov/pmc/articles/PMC4200225/). An overview of the analysed species and strains, their origin and their accession numbers is shown in Table 1.

### Data management and integration

Genomic data was modeled using the Django object relational mapper (ORM) (http://www.djangoproject.com) and database tables were automatically created using the management command ‘*syncdb*’. The FASTA protein sequences of putative outer membrane proteins (OMP) of the selected strains of *E*. *coli*, *Campylobacter* and *Helicobacter* were extracted from the genomes by using the HHomp tool^22^ as described before^15^. The strains of which their corresponding genome sequences are fully known were deposited in the EMBL databases and the accession numbers are shown in the tables. From the other strains, with unknown genome sequences, their OMPs were uploaded in the database. Subsequently, all OMPs were combined to a single ‘*combined*.*fasta*’ file using the Unix ‘*cat*’ command. Next, a Phython script was written using IPhython Notebook (http://ipython.org/notebook.html), to load all sequences to the database.

### Classification into OMP families

The seed alignments from the 90 OMP families that are defined in OMPdb.org were downloaded. For each of these families, the seed alignments were converted to HMM profiles using the HMMER3 suite of programs (http://hmmer.janelia.org). Specifically, the ‘*hmmbuild’* program was executed for each seed alignment as follows: ‘*hmmbuild* <*hmmfile_out*> <*seed_alignment*>*’*. The generated HMM profiles were then stored to the database. Next, the HMM profiles for all of the OMP families were collected and “pressed” for faster searches using the program ‘*hmmpress’* in HMMER3, resulting in an HMM database of OMP families. Then, each OMP protein sequence was searched against this HMM database in order to classify them into any of those OMP families. The remaining unclassified proteins were searched against the Pfam database using ‘*hmmscan*’ in the HMMER3 web server.

Finally, the last remaining 278 unclassified proteins were clustered into groups with CD-HIT using the settings ‘word length = 2, identity cutoff = 40%’ in order to produce the fewest possible number of clusters. The clusters with single members with an amino acid length of <120 (52 sequences in total) were dropped. The distribution of the OMP families from the *Escherichia*-, *Campylobacter*- and *Helicobacter* genera was determined with ‘*pandas*’, a data processing package in Python (http://pandas.pydata.org). For plotting and visualization, the ‘*matplotlib’* plotting package was used (http://matplotlib.org).

### Alignment and phylogenetic analysis

The OMP families with at least 3 identified members were subjected to phylogenetic analysis. The OMP protein sequences of these families were written into multi-sequence FASTA files (one for each family). Multiple sequence alignment was performed using the Clustal Omega program (http://www.clustal.org/omega/). The alignments were trimmed with ‘TrimAl’ program (http://trimal.cgenomics.org) in order to remove sequence regions that potentially blur the phylogenetic signal such as highly variable loop regions. The trimmed alignments were then fed into the ‘FastTree’ phylogenetic tree-building program (http://www.microbesonline.org/fasttree). The phylogenetic trees were visualized and edited by the online tool ‘Interactive Tree Of Life’ (iTol)^66^. The best-fitting root was selected with TempEst v1.5^67^, formerly known as ‘Path-O-Gen’. The OMP protein sequences of all species and strains were subjected to protein BLAST (ncbi). In this way, the individual OMPs belonging to each family as well as possible subgroups could be distinguished. The relative positions of all species’ OMPs in the phylogenetic trees were studied and evaluated, and the possible roles in virulence and colonization are presented.

### Accession codes

The NCBI or EMBL accession numbers of the species and strains with fully annotated genomes are provided in Table 1.

## Electronic supplementary material

Supplementary dataset
