# Supplementary material for: In silico proteomic and phylogenetic analysis of the outer membrane protein repertoire of gastric Helicobacter species
Source: Sci Rep. 2018 Oct 18;8:15453. doi: 10.1038/s41598-018-32476-1 (PMC6194013; doi:10.1038/s41598-018-32476-1)
Supplement: Supplementary file 1 — Supplementary dataset [file 41598_2018_32476_MOESM1_ESM.pdf]

In silico proteomic and phylogenetic analysis of the outer membrane protein repertoire of gastric *Helicobacter* species

Eva Bauwens[1$], Myrthe Joosten[1$], Joemar Taganna[2,3,#], Mirko Rossi[4,#], Ayla Debraekeleer[2,3], Alfred Tay[5], Fanny Peters[5], Steffen Backert[6], James Fox[7], Richard Ducatelle[1], Han Remaut[2,3,+], Freddy Haesebrouck[1,*,+] and Annemieke Smet[1,8,+]

[1]Department of Pathology, Bacteriology and Avian Diseases, Faculty of Veterinary Medicine, Ghent University, Merelbeke, Belgium; [2]Laboratory of Structural and Molecular Microbiology, Structural Biology Research Center, Flemish Institute for Biotechnology (VIB), Brussels, Belgium; [3]Structural Biology Brussels, Free University of Brussels (VUB), Brussels, Belgium; [4]Department of Food Hygiene and Environmental Health, Faculty of Veterinary Medicine, University of Helsinki, Helsinki, Finland; [5]The Marshall Centre for Infectious Diseases Research and Training, School of Pathology and Laboratory Medicine, University of Western Australia, Nedlands, Perth, Western Australia, Australia; [6]University Erlangen Nürnberg, Department Biology, Division Microbiology, Erlangen, Germany; [7]Division of Comparative Medicine, Massachusetts Institute of Technology, Cambridge, MA, USA; [8]Laboratorium of Experimental Medicine and Pediatrics, Faculty of Medicine and Health Sciences, University of Antwerp, Antwerp, Belgium

*Address correspondence to Freddy Haesebrouck, (freddy.haesebrouck@ugent.be)

$ E. B. and M. J. contributed equality to this work (shared first author)

# J. T. and M. R. contributed equally to this work (shared second author)

+ H. R., F. H. and A. S. contributed equally to this work (shared senior authorship)

Table S1. Overview of the 90 OMP families from the OMPdb database

OMP		*E. coli*					*C. coli*			*C. jejuni*			*H. acinonychis*				*H. ailurogastricus*				*H. baculiformis*	*H. hepaticus*	*H. bizzozeronii*				*H. cetorum*		*H. cinaedi*	*H. cynogastricus*	*H. equorum*	*H. felis*				*H. heilmannii*					*H. mustelae*	*H. pylori*						*H. salomonis*			*H. suis*				*H. trogontum*
		536	IAI1	IAI39	K12-W3110	TW14359	15-157360	76339	N29710	00-2425	4031	M1	Hacino1	Hacino2	Hacino3	Hacino4	ASB7	ASB9	ASB11	ASB13	M50	ATC 51449	10	14	CIII	M7	MIT 00-7128	MIT 99-5656	BAA_847	JKM4	eqF1	CS1	CS6	CS7	DS1	ASB1	ASB2	ASB3	ASB6	ASB14	ATC12198	Puno120	G27	F30	J99	India7	SouthAfrica7	M45	R1053	KokIII	HS2	HS4	HS7	HS9	R3554
	NCBI accession number	NC_008253	NC_011741	NC_011750	NC_007779	NC_013008	NC_022660	NC_022132	NC_022347	NC_022362	NC_022529	NC_017280	FZMD00000000	FZLX00000000	NC_008229	FZLX00000000	CDMG00000000	CDMN00000000	CDML00000000	CDMH00000000	FZMF00000000	NC_004917	FZEH00000000	FZMK00000000	NC_015674	FZLK00000000	NC_017737	NC_017735	NC_020555	FZMQ00000000	FZPO00000000	NC_014810	FZKM00000000	FZKX00000000	FZNI00000000	CDMK00000000	CDMP00000000	CDMJ00000000	CDMM00000000	CDMI00000000	NC_013949	NC_017378	NC_011333	NC_017365	NC_000921	NC_017372	NC_022130	FZLZ00000000	OANQ00000000	FZMA00000000	FZLI00000000	FZKI00000000	FZKH00000000	FZLE00000000	FZNG00000000
1	PagL	×	×	×	×	×	×															×																																	
2	OmpIP/ Omp85	×*	×*	×*	×*	×*	×	×	×	×	×	×	×	×	×	×	×	×*	×	×	×	×	×	×	×	×	×	×	×	×	×	×	×	×	×	×	×	×	×	×	×*	×	×	×	×			×	×	×	×	×	×	×	×
3	OMR-TonB	×*	×*	×*	×*	×*	×*	×*	×*	×*	×	×	×*	×*	×*	×*	×	×	×	×	×	×*					×*	×*	×*	×	×*	×	×	×	×	×*	×	×	×	×	×*	×*	×*	×*	×*	×*	×*				×*	×	×*	×*	×*
4	OprB																																																						
5	OmpG																																																						
6	TPS	×				×		×*	×																																														
7	AT	×*	×*	×*	×*	×*	×*	×*	×*	×	×*	×*																×*			×										×*		×	×											×*
8	OmpX					×																																																	
9	GBP-1	×	×*	×*	×	×																	×		×																														
10	IBSA			×*																																																			
11	FUP	×*	×*	×*	×*	×*																																																	
12	FadL						×	×	×				×	×	×	×	×*	×*	×*	×	×*	×	×	×	×*	×	×	×*	×	×		×*	×*	×*	×*	×	×	×	×	×	×	×	×	×	×	×		×	×	×	×	×	×	×	×*
13	HOP-1							×		×			×*	×*	×*	×*	×*	×*	×*	×*	×*	×*	×*	×*	×*	×*	×*	×*	×*	×*	×*	×*	×*	×*	×*	×*	×*	×*	×*	×*	×*	×*	×*	×*	×*	×*	×*	×*	×*	×*	×*	×*	×*	×*	×*
14	Imp/ OstA						×	×	×	×	×	×	×	×	×	×	×	×	×	×	×	×	×	×	×	×	×	×	×	×	×	×	×	×	×	×	×	×	×	×	×	×	×	×	×	×	×	×	×	×	×	×	×	×	
15	GBP-2																																																						

OMP		*E. coli*					*C. coli*			*C. jejuni*			*H. acinonychis*				*H. ailurogastricus*				*H. baculiformis*	*H. hepaticus*	*H. bizzozeronii*				*H. cetorum*		*H. cinaedi*	*H. cynogastricus*	*H. equorum*	*H. felis*				*H. heilmannii*					*H. mustelae*	*H. pylori*						*H. salomonis*			*H. suis*				*H. trogontum*
		536	IAI1	IAI39	K12-W3110	TW14359	15-157360	76339	N29710	00-2425	4031	M1	Hacino1	Hacino2	Hacino3	Hacino4	ASB7	ASB9	ASB11	ASB13	M50	ATC 51449	10	14	CIII	M7	MIT 00-7128	MIT 99-5656	BAA_847	JKM4	eqF1	CS1	CS6	CS7	DS1	ASB1	ASB2	ASB3	ASB6	ASB14	ATC12198	Puno120	G27	F30	J99	India7	SouthAfrica7	M45	R1053	KokIII	HS2	HS4	HS7	HS9	R3554
	NCBI accession number	NC_008253	NC_011741	NC_011750	NC_007779	NC_013008	NC_022660	NC_022132	NC_022347	NC_022362	NC_022529	NC_017280	FZMD00000000	FZLX00000000	NC_008229	FZLX00000000	CDMG00000000	CDMN00000000	CDML00000000	CDMH00000000	FZMF00000000	NC_004917	FZEH00000000	FZMK00000000	NC_015674	FZLK00000000	NC_017737	NC_017735	NC_020555	FZMQ00000000	FZPO00000000	NC_014810	FZKM00000000	FZKX00000000	FZNI00000000	CDMK00000000	CDMP00000000	CDMJ00000000	CDMM00000000	CDMI00000000	NC_013949	NC_017378	NC_011333	NC_017365	NC_000921	NC_017372	NC_022130	FZLZ00000000	OANQ00000000	FZMA00000000	FZLI00000000	FZKI00000000	FZKH00000000	FZLE00000000	FZNG00000000
16	OmpT	×		×	×	×																																																			
17	OmpA	×	×*	×*	×	×*		×	×	×	×	×																																											
18	NspA	×*	×		×																×										×																×								
19	OprD	×	×	×	×	×																																																	
20	OprF Porin																															×	×	×	×																				
21	OmpW					×																×*																										×	×	×	×	×	×	×	
22	POP																																																						
23	MipA/ OmpV		×	×	×	×																																																	
24	KdgM																																																						
25	YfaZ OMP	×	×*	×	×	×																																																	
26	Tsx	×*	×	×*	×*	×																																																	
27	OpcA																																																						
28	CopB																																																						
29	SspA	×*	×	×	×	×																×																																	
30	GBP-4	×*	×*	×*	×*	×*																																																	
31	SP	×*	×*	×*	×*	×																																																	

OMP		*E. coli*					*C. coli*			*C. jejuni*			*H. acinonychis*				*H. ailurogastricus*				*H. baculiformis*	*H. hepaticus*	*H. bizzozeronii*				*H. cetorum*		*H. cinaedi*	*H. cynogastricus*	*H. equorum*	*H. felis*				*H. heilmannii*					*H. mustelae*	*H. pylori*						*H. salomonis*			*H. suis*				*H. trogontum*
		536	IAI1	IAI39	K12-W3110	TW14359	15-157360	76339	N29710	00-2425	4031	M1	Hacino1	Hacino2	Hacino3	Hacino4	ASB7	ASB9	ASB11	ASB13	M50	ATC 51449	10	14	CIII	M7	MIT 00-7128	MIT 99-5656	BAA_847	JKM4	eqF1	CS1	CS6	CS7	DS1	ASB1	ASB2	ASB3	ASB6	ASB14	ATC12198	Puno120	G27	F30	J99	India7	SouthAfrica7	M45	R1053	KokIII	HS2	HS4	HS7	HS9	R3554
	NCBI accession number	NC_008253	NC_011741	NC_011750	NC_007779	NC_013008	NC_022660	NC_022132	NC_022347	NC_022362	NC_022529	NC_017280	FZMD00000000	FZLX00000000	NC_008229	FZLX00000000	CDMG00000000	CDMN00000000	CDML00000000	CDMH00000000	FZMF00000000	NC_004917	FZEH00000000	FZMK00000000	NC_015674	FZLK00000000	NC_017737	NC_017735	NC_020555	FZMQ00000000	FZPO00000000	NC_014810	FZKM00000000	FZKX00000000	FZNI00000000	CDMK00000000	CDMP00000000	CDMJ00000000	CDMM00000000	CDMI00000000	NC_013949	NC_017378	NC_011333	NC_017365	NC_000921	NC_017372	NC_022130	FZLZ00000000	OANQ00000000	FZMA00000000	FZLI00000000	FZKI00000000	FZKH00000000	FZLE00000000	FZNG00000000
32	MomP							x																																															
33	HOP-2												x*	x*	x*	x*	x*	x*	x*	x*	x*	x*	x*	x*	x*	x*	x*	x*	x*	x*	x*	x*	x*	x*	x*	x*	x*	x*	x*	x*	x*	x*	x*	x*	x*	x*	x*	x*	x*	x*	x*	x*	x*	x*	x*
34	TSA																																																						
35	OMP β-bd	x*	x	x*	x*	x*		x		x	x	x	x*	x*	x*	x*	x*	x*	x*	x*	x*	x*	x*	x*	x*	x*	x*	x*	x*	x*	x*	x*	x*	x*	x*	x*	x*	x*	x*	x*	x*	x*	x*	x*	x*	x*	x*	x*	x*	x*	x*	x*	x*	x*	x*
36	SfpA/ LpxR					x							x	x	x	x	x	x	x	x	x*		x*	x*	x*	x*	x	x		x*		x*	x*	x*	x*	x*	x*	x*	x	x	x	x	x	x	x	x	x	x*	x*	x*	x	x	x	x	x
37	PagP	x	x	x	x	x																																																	
38	OMPLA	x	x	x	x	x	x	x	x	x	x	x	x	x	x	x	x	x	x	x	x	x	x	x	x	x		x	x	x		x	x	x	x	x	x	x	x	x		x		x	x	x	x	x	x	x	x	x	x	x	x*
39	BP-p13																																																						
40	BP-Oms28																																																		x	x	x	x	
41	BcsC	x	x	x	x	x																																																	
42	OMF	x*	x*	x*	x*	x*	x*	x*	x*	x*	x*	x*	x*	x*	x*	x*	x*	x*	x*	x*	x*	x*	x*	x*	x*	x*	x*	x*	x*	x*	x*	x*	x*	x*	x*	x*	x*	x*	x*	x*	x*	x*	x*	x*	x*	x*	x*	x*	x*	x*	x*	x*	x*	x*	x*
43	BRP																																																						
44	AT-2	x*	x*	x*	x*	x*																																																	x
45	Secretin	x*	x*	x*	x*	x*	x	x	x	x	x	x										x							x		x																								x
46	SH-VSP																																																						
47	TP-MSP	x	x	x	x	x															x															x	x	x	x	x															

OMP		*E. coli*					*C. coli*			*C. jejuni*			*H. acinonychis*				*H. ailurogastricus*				*H. baculiformis*	*H. hepaticus*	*H. bizzozeronii*				*H. cetorum*		*H. cinaedi*	*H. cynogastricus*	*H. equorum*	*H. felis*				*H. heilmannii*					*H. mustelae*	*H. pylori*						*H. salomonis*			*H. suis*				*H. trogontum*
		536	IAI1	IAI39	K12-W3110	TW14359	15-157360	76339	N29710	00-2425	4031	M1	Hacino1	Hacino2	Hacino3	Hacino4	ASB7	ASB9	ASB11	ASB13	M50	ATC 51449	10	14	CIII	M7	MIT 00-7128	MIT 99-5656	BAA_847	JKM4	eqF1	CS1	CS6	CS7	DS1	ASB1	ASB2	ASB3	ASB6	ASB14	ATC12198	Puno120	G27	F30	J99	India7	SouthAfrica7	M45	R1053	KokIII	HS2	HS4	HS7	HS9	R3554
	NCBI accession number	NC_008253	NC_011741	NC_011750	NC_007779	NC_013008	NC_022660	NC_022132	NC_022347	NC_022362	NC_022529	NC_017280	FZMD00000000	FZLX00000000	NC_008229	FZLX00000000	CDMG00000000	CDMN00000000	CDML00000000	CDMH00000000	FZMF00000000	NC_004917	FZEH00000000	FZMK00000000	NC_015674	FZLK00000000	NC_017737	NC_017735	NC_020555	FZMQ00000000	FZPO00000000	NC_014810	FZKM00000000	FZKX00000000	FZNI00000000	CDMK00000000	CDMP00000000	CDMJ00000000	CDMM00000000	CDMI00000000	NC_013949	NC_017378	NC_011333	NC_017365	NC_000921	NC_017372	NC_022130	FZLZ00000000	OANQ00000000	FZMA00000000	FZLI00000000	FZKI00000000	FZKH00000000	FZLE00000000	FZNG00000000
48	*Borrelia* Oms66/ Omp66																																																									
49	CP																																																									
50	LP-OmpL1																																																									
51	*Camp.* omp50										x																		x																													
52	FT- fslE																																																									
53	LP-MOMP																																																									
54	Intimin/Invasin	x*	x*	x*	x*	x*	x	x	x	x	x	x																																												
55	nfrA																																																									
56	wzi																																																									
57	algE																																																									
58	TT- HB27 TtoA																																																									
59	AB 34-kDa OMP																																																									
60	CymA		x																																																							
61	fomA																																																									
62	carO																																																									

OMP		*E. coli*					*C. coli*			*C. jejuni*			*H. acinonychis*				*H. ailurogastricus*				*H. baculiformis*	*H. hepaticus*	*H. bizzozeronii*				*H. cetorum*		*H. cinaedi*	*H. cynogastricus*	*H. equorum*	*H. felis*				*H. heilmannii*					*H. mustelae*	*H. pylori*						*H. salomonis*			*H. suis*				*H. trogontum*
		536	IAI1	IAI39	K12-W3110	TW14359	15-157360	76339	N29710	00-2425	4031	M1	Hacino1	Hacino2	Hacino3	Hacino4	ASB7	ASB9	ASB11	ASB13	M50	ATC 51449	10	14	CIII	M7	MIT 00-7128	MIT 99-5656	BAA_847	JKM4	eqF1	CS1	CS6	CS7	DS1	ASB1	ASB2	ASB3	ASB6	ASB14	ATC12198	Puno120	G27	F30	J99	India7	SouthAfrica7	M45	R1053	KokIII	HS2	HS4	HS7	HS9	R3554
	NCBI accession number	NC_008253	NC_011741	NC_011750	NC_007779	NC_013008	NC_022660	NC_022132	NC_022347	NC_022362	NC_022529	NC_017280	FZMD00000000	FZLX00000000	NC_008229	FZLX00000000	CDMG00000000	CDMN00000000	CDML00000000	CDMH00000000	FZMF00000000	NC_004917	FZEH00000000	FZMK00000000	NC_015674	FZLK00000000	NC_017737	NC_017735	NC_020555	FZMQ00000000	FZPO00000000	NC_014810	FZKM00000000	FZKX00000000	FZNI00000000	CDMK00000000	CDMP00000000	CDMJ00000000	CDMM00000000	CDMI00000000	NC_013949	NC_017378	NC_011333	NC_017365	NC_000921	NC_017372	NC_022130	FZLZ00000000	OANQ00000000	FZMA00000000	FZLI0000000	FZKI00000000	FZKH00000000	FZLE00000000	FZNG00000000
63	mspA																																																						
64	*Geob.* ompJ																																																						
65	STY4528																																																							
66	yaiO OMP	x*	x*	x*	x*	x																																																	
67	Oms38 Spirochaetes																																																							
68	hmw1B																																																							
69	*Aggre. actino.*mp 67/ morC	x	x	x	x	x																																																	
70	rafY	x*		x*		x																																																	
71	SAP																																																							
72	MOMP/OmpA																										x	x																x											
73	DUF 1597	x	x	x	x	x																																																	
74	DUF 3308																													x											x														
75	PG- PorT	x	x	x*	x	x											x*	x*	x*	x*		x	x*		x	x	x*	x*		x*		x*	x*	x*	x	x*	x	x*	x*	x		x		x*	x*	x		x*	x*	x*					x*
76	DUF 2320																																																						
77	DUF 3374		x		x																																																		

OMP		*E. coli*					*C. coli*			*C. jejuni*			*H. acinonychis*				*H. ailurogastricus*				*H. baculiformis*	*H. hepaticus*	*H. bizzozeronii*				*H. cetorum*		*H. cinaedi*	*H. cynogastricus*	*H. equorum*	*H. felis*				*H. heilmannii*					*H. mustelae*	*H. pylori*						*H. salomonis*			*H. suis*				*H. trogontum*
		536	IAI1	IAI39	K12-W3110	TW14359	15-157360	76339	N29710	00-2425	4031	M1	Hacino1	Hacino2	Hacino3	Hacino4	ASB7	ASB9	ASB11	ASB13	M50	ATC 51449	10	14	CIII	M7	MIT 00-7128	MIT 99-5656	BAA_847	JKM4	eqF1	CS1	CS6	CS7	DS1	ASB1	ASB2	ASB3	ASB6	ASB14	ATC12198	Puno120	G27	F30	J99	India7	SouthAfrica7	M45	R1053	KokIII	HS2	HS4	HS7	HS9	R3554
	NCBI accession number	NC_008253	NC_011741	NC_011750	NC_007779	NC_013008	NC_022660	NC_022132	NC_022347	NC_022362	NC_022529	NC_017280	FZMD00000000	FZLX00000000	NC_008229	FZLX00000000	CDMG00000000	CDMN00000000	CDML00000000	CDMH00000000	FZMF00000000	NC_004917	FZEH00000000	FZMK00000000	NC_015674	FZLK00000000	NC_017737	NC_017735	NC_020555	FZMQ00000000	FZPO00000000	NC_014810	FZKM00000000	FZKX00000000	FZNI00000000	CDMK00000000	CDMP00000000	CDMJ00000000	CDMM00000000	CDMI00000000	NC_013949	NC_017378	NC_011333	NC_017365	NC_000921	NC_017372	NC_022130	FZLZ00000000	OANQ00000000	FZMA00000000	FZLI00000000	FZKI00000000	FZKH00000000	FZLE00000000	FZNG00000000
78	OMP beta-barrel	x	x*		x*	x*	x*		x*		x*	x*															x		x*	x			x								x														
79	TraF					x														x	x	x*		x		x	x		x*		x*																	x	x	x					x
80	DUF 2490	x	x	x	x	x	x		x	x	x	x																																											
81	DUF 3575																				x	x		x			x			x		x	x	x	x													x	x	x					
82	DUF 2860																																																						
83	DUF 4289																																																						
84	DUF 3078																																																						
85	DUF 3138																																																						
86	DUF 560			x			x	x	x	x	x	x																																											
87	Putative MetA-POPD	x																																																					
88	DUF 1302																																																						
89	DUF 3187																														x																								
90	Gcw_chp																																																						

Shown are the OMP family names (left column) and the strain names of *E. coli* and the *Campylobacter* and *Helicobacter* species with homologous OMP sequences (top row). Strains with multiple OMP sequences belonging to the same family are indicated with an asterisk *. "x" indicates that the protein is present. (**1. PagL** = outer membrane-localized lipid A 3-O-deacylase, function: enzyme; **2. OmpIP/Omp85** = Outer Membrane Protein Insertion porin, function: OMP biogenesis/secretion; **3. OMR-TonB** = Outer Membrane Receptor Dependent Receptor, function: receptor; **4. OprB** = Glucose-selective OprB Porin, function: specific channels; **5. OmpG** = OmpG Porin, function: non-specific channels; **6. TPS** = Two-Partner Secretion, function: biogenesis/secretion; **7. AT** = Autotransporter, function: biogenesis/secretion; **8. OmpX** = Outer Membrane Protein X, function: adhesion; **9. GBP-1** =

General Bacterial Porin Family 1, function: non-specific channels; **10. IBSA** = Intracellular Bacteria Surface Antigen, function: adhesion; **11. FUP** = Outer Membrane Fimbrial Usher Porin, function: pilus biogenesis; **12. FadL** = FadL Outer Membrane Protein, function: receptor; **13. HOP-1** = *Helicobacter* Outer Membrane Protein Family 1, function: adhesion; **14. Imp/OstA**, function: biogenesis/secretion; **15. GBP-2** = General Bacterial Porin Family 2, functioin: non-specific channels; **16. OmpT** = Omptin, function: enzyme; **17. OmpA**, function: structural; **18. NspA** = Neisserial Surface Protein A, function: adhesion; **19. OprD** = Outer Membrane Porin, function: specific channels; **20. OprF porin**, function: structural; **21. OmpW**, function: non-specific channels; **22. POP** = *Pseudomonas* OprP Porin, function: specific channels; **23. MipA/OmpV**, function: structural; **24. KdgM** = Oligogalucturonate-specific Porin, function: specific channels; **25. YfaZ OMP** = YfaZ Outer Membrane Protein, function: unknown; **26. Tsx** = Nucleoside-specific Channel-forming Outer Membrane Porin, function: receptor; **27. Opc** = opacity, function: adhesion; **28. CopB** = Copper resistance protein B, function: specific channels; **29. SspA** = Salt-stress induced outer membrane protein, function: unknown; **30. GBP-4** = General Bacterial Porin Family 4, function: non-specific channels; **31. SP** = Sugar Porin, function: specific channels; **32. MomP** = *Campylobacter jejuni* Major Outer Membrane Porin, function: non-specific channels; **33. HOP-2** = *Helicobacter* Outer Membrane Protein Family 2; **34. TSA** = Type Specific Antigen, function: unknown; **35. OMP β-bd** = Outer Membrane Protein beta-barrel domain, function: unknown; **36. SfpA/LpxR** = Systemic factor protein A, function: enzyme; **37. PagP** = Antimicrobial peptide resistance and lipid A acylation protein, function: enzyme; **38. OMPLA** = Outer Membrane Phospholipase, function: enzyme; **39. BP-p13** = *Borrelia Porin* p13, function: non-specific channels; **40. BP-oms28** = *Borrelia* Oms28 porin, function: non-specific channels; **41. BcsC** = Bacterial Cellulose Synthase Operon Protein C, function: biogenesis/secretion; **42. OMF** = Outer Membrane Factor, function: biogenesis/secretion; **43. BRP** = *Brucella*-Rhizobium Porin, function: non-specific channels; **44. AT-2** = Autotransporter-2, function: biogenesis/secretion; **45. Secretin**, function: biogenesis/secretion; **46. SH-VSP** = *Serpulina hyodysenteriae* variable surface protein, function: unknown; **47. TP-MSP** = *Treponema* Porin Major Surface Protein, function: adhesion; **48. Borrelia Oms66/Omp66**, function: non-specific channels; **49. CP** = Chlamydial Porin, function: non-specific channels; **50. LP-OmpL1** = *Leptospira* Porin OmpL1, function: non-specific channels; **51. *Camp* omp50** = *Campylobacter* omp50, function: non-specific channels; **52. FT- fslE** = *Francisella tularensis* fslE, function: receptor; **53. LP-MOMP** = *Legionella pneumophila* major outer membrane protein, function: adhesion; **54. Intimin/Invasin**, function: adhesion; **55. nfrA** = N4 bacteriophage Receptor, function: unkown; **56. wzi**, function: biogenesis/secretion; **57. algE** = Alginate Export Porin, function: unknown; **58. TT- HB27 TtoA** = *Thermus thermophilus* HB27 TtoA, function: unknown; **59. AB 34-kDa OMP** = *Acinetobacter baumannii* 34-kDa outer membrane protein, function: specific channels; **60. CymA** = Cyclodextrin Porin, function: specific channels; **61. fomA** = Fusobacterial Outer Membrane Porin, function: non-specific channels; **62. carO** = Carbapenem resistance-associated outer membrane protein, function: specific channels; **63. mspA** = *Treponema* Major outer membrane protein, function: unknown; **64. Geob. ompJ** = *Geobacter* ompJ, function: structural; **65. STY4528** = *Salmonella typhi* STY4528, function: unknown; **66. yaiO OMP** = yaiO Outer Membrane Protein, function: unknown; **67. Oms38 Spirochaetes**, function: unknown; **68. hmw1B** = *Haemophilus influenza* Outer Membrane Translocator, function: biogenesis/secretion; **69. Aggre.actino.mp67/morC** = *Aggregatibacter actinomycetemcomitans* omp67/morC, function: biogenesis/secretion; **70. rafY** = Raffinose Porin, function: specific channels; **71. SAP** = Short Chain Amide and Urea Porin, function: specific channels; **72. MOMP/OmpA** = *Porphyromonas* Major Outer Membrane, function: structural; **73. DUF1597**, function: unknonwn; **74. DUF3308**, function: unknown; **75. PG- PorT** = *Porphyromonas gingivalis* PorT, function: biogenesis/secretion; **76. DUF2320**, function: unknown; **77. DUF3374**, function: unknown; **78. OMP beta-barrel** = Outer membrane protein beta-barrel, function: unknown; **79. TraF** = F plasmid transfer operon, function: unknown; **80. DUF2490**, function: unknown; **81. DUF3575**, function: unknown; **82. DUF2860**, function: unknown; **83. DUF4289**, function: unknown; **84. DUF3078**, function: unknown; **85. DUF3138**, function: unknown; **86. DUF560**, function: unknown; **87. Putative MetA-POPD** = Putative MetA-pathway of phenol degradation, function: unknown; **88. DUF1302**, function: unknown; **89.DUF3187**, function: unknown; **90. Gcw_chp**, function: unknown.

Table S2. Family x1-x31 from the Pfam database

X		*E. coli*					*C. coli*			*C. jejuni*			*H. acinonychis*				*H. ailurogastricus*				*H. baculiformis*	*H. hepaticus*	*H. bizzozeronii*				*H. cetorum*		*H. cinaedi*	*H. cynogastricus*	*H. equorum*	*H. felis*				*H. heilmannii*					*H. mustelae*	*H. pylori*						*H. salomonis*			*H. suis*				*H. trogontum*
		536	IAI1	IAI39	K12-W3110	TW14359	15-157360	76339	N29710	00-2425	4031	M1	Hacino1	Hacino2	Hacino3	Hacino4	ASB7	ASB9	ASB11	ASB13	M50	ATC 51449	10	14	CIII	M7	MIT 00-7128	MIT 99-5656	BAA_847	JKM4	eqF1	CS1	CS6	CS7	DS1	ASB1	ASB2	ASB3	ASB6	ASB14	ATC12198	Puno120	G27	F30	J99	India7	SouthAfrica7	M45	R1053	KokIII	HS2	HS4	HS7	HS9	R3554
	NCBI accession number	NC_008253	NC_011741	NC_011750	NC_007779	NC_013008	NC_022660	NC_022132	NC_022347	NC_022362	NC_022529	NC_017280	FZMD00000000	FZLX00000000	NC_008229	FZLX00000000	CDMG00000000	CDMN00000000	CDML00000000	CDMH00000000	FZMF00000000	NC_004917	FZEH00000000	FZMK00000000	NC_015674	FZLK00000000	NC_017737	NC_017735	NC_020555	FZMQ00000000	FZPO00000000	NC_014810	FZKM00000000	FZKX00000000	FZNI00000000	CDMK00000000	CDMP00000000	CDMJ00000000	CDMM00000000	CDMI00000000	NC_013949	NC_017378	NC_011333	NC_017365	NC_000921	NC_017372	NC_022130	FZLZ00000000	OANQ00000000	FZMA00000000	FZLI00000000	FZKI00000000	FZKH00000000	FZLE00000000	FZNG00000000
1	Vac												x*	x*	x*	x*											x	x														x	x	x	x	x	x								
2	TonB-DRPL	x	x	x	x*	x							x*	x*	x*	x*	x*	x*	x*	x*	x*	x	x*	x*	x*	x*	x*	x	x*	x	x	x	x	x	x	x*	x*	x*	x*	x*	x*	x*	x*	x*	x*	x*	x*				x*	x*	x*	x*	x*
3	Putative Vac												x*	x*	x*	x*	x	x	x	x	x			x	x	x	x*	x*		x		x	x	x	x	x	x	x	x	x		x*	x*	x*	x*	x*	x*	x	x	x	x	x	x	x	x
4	DUF2622	x	x	x	x	x																																																	
5	Sel1 repeat																						x	x	x	x*						x	x	x	x																				
6	AhpC/ TSA	x	x	x	x	x																																																	
7	PapC C-TD		x																																																				
8	TTP repeat				x	x																																																	
9	DUF940	x	x*	x	x*	x*																																																	
10	PSB/EP	x	x*	x*	x*	x*				x	x	x																																											
11	Fimbrial protein		x		x	x																																																	
12	GSS pre ATPgrasp																																x																						
13	FimH	x	x	x	x	x																																																	
14	SSP-B	x	x	x	x	x																																																	
15	Asp/Orn CT																																								x														

X		*E. coli*					*C. coli*			*C. jejuni*			*H. acinonychis*				*H. ailurogastricus*				*H. baculiformis*	*H. hepaticus*	*H. bizzozeronii*				*H. cetorum*		*H. cinaedi*	*H. cynogastricus*	*H. equorum*	*H. felis*				*H. heilmannii*					*H. mustelae*	*H. pylori*						*H. salomonis*			*H. suis*				*H. trogontum*
		536	IAI1	IAI39	K12-W3110	TW14359	15-157360	76339	N29710	00-2425	4031	M1	Hacino1	Hacino2	Hacino3	Hacino4	ASB7	ASB9	ASB11	ASB13	M50	ATC 51449	10	14	CIII	M7	MIT 00-7128	MIT 99-5656	BAA_847	JKM4	eqF1	CS1	CS6	CS7	DS1	ASB1	ASB2	ASB3	ASB6	ASB14	ATC12198	Puno120	G27	F30	J99	India7	SouthAfrica7	M45	R1053	KokIII	HS2	HS4	HS7	HS9	R3554
	NCBI accession number	NC_008253	NC_011741	NC_011750	NC_007779	NC_013008	NC_022660	NC_022132	NC_022347	NC_022362	NC_022529	NC_017280	FZMD00000000	FZLX00000000	NC_008229	FZLX00000000	CDMG00000000	CDMN00000000	CDML00000000	CDMH00000000	FZMF00000000	NC_004917	FZEH00000000	FZMK00000000	NC_015674	FZLK00000000	NC_017737	NC_017735	NC_020555	FZMQ00000000	FZPO00000000	NC_014810	FZKM00000000	FZKX00000000	FZNI00000000	CDMK00000000	CDMP00000000	CDMJ00000000	CDMM00000000	CDMI00000000	NC_013949	NC_017378	NC_011333	NC_017365	NC_000921	NC_017372	NC_022130	FZLZ00000000	OANQ00000000	FZMA00000000	FZLI00000000	FZKI00000000	FZKH00000000	FZLE00000000	FZNG00000000
16	Fibro type III					x*																																																	
17	POTRA-domain					x				x	x*	x*																																											
18	OstA-like protein																																																						x
19	PAPP-A																											x																											
20	Transposase	x																																																					
21	ESP of type V SS			x	x	x																																																	
22	Bact. Ig-like dom.	x																																																					
23	TTTF receptor						x		x																																														
24	CBS GfcC					x																																																	
25	DUF997	x																																																						
26	SLBB domain							x	x																																														
27	tRNA SC2CD																									x																														
28	Peptidyl-tRNA hyd																									x																														
29	P&F-AC, PapD N				x																																																		
30	ATP		x	x	x	x																																																	
31	E. protein Yae1																																								x														

Shown are the OMP family names (left column) and the strain names of *E. coli* and *Helicobacter* species with homologous OMP 879 sequences (top row). Strains with multiple OMP sequences belonging to the same family are indicated with an asterisk *. "x" indicates that the protein is present. In general, most families were present only in 1 species and often not in all analyzed strains. (**1. Vac** = vacuolating cytotoxin; **2. TonB-DRPL** = TonB-dependent Receptor Plug Domain; **3. Putative Vac** = Putative vacuolating cytotoxin; **4. DUF2622** = protein of unknown function; **5. Sel1 repeat**; **6. AhpC/TSA** = AhpC/TSA family; **7. PapC C-TD** = PapC C-terminal domain; **8. TTP repeat** = Tetratricopeptide repeat; **9. DUF940** = bacterial putative lipoprotein; **10. PSB/EP** = Polysaccharide biosynthesis/export protein; **11. Fimbrial protein**; **12. GSS pre ATPgrasp** = Glutathionylspermidine synthase preATP-grasp; **13. FimH** = FimH, mannose binding; **14. SSP-B** = Stringent starvation protein B; **15. Asp/Orn CT** = Aspartate/ornithine carbamoyltransferase, carbamoyl-P binding domain; **16. Fibro type III** = Fibronectin type III protein; **17. POTRA-domain** = POTRA domain, ShlB-type; **18. OstA-like protein**; **19. PAPP-A** = Pregnancy-associated plasma protein-A; **20. Transposase**; **21. ESP of type V SS** = Extended Signal Peptide of Type V secretion system; **22. Bact. Ig-like dom.** = Bacterial Ig-like domain (group 1); **23. TTTF receptor** = Tripartite tricarbocylate transporter family receptor; **24. CBS GfcC** = Capsule biosynthesis GfcC; **25. DUF997** = protein of unknown function; **26. SLBB domain**; **27. tRNA SC2CD** = tRNA synthetase class II core domain (G,H,P,S and T); **28. Peptidyl-tRNA hyd** = Peptidyl-tRNA hydrolase; **29. P&F-AC, PapD N** = Pili and flagellar-assembly chaperone, PapD N-terminal domain; **30. ATP** = corinoid adenosyltransferase BtuR/CobO/CobP; **31. E. protein Yae1** = Essential protein Yae1, N terminal.

Table S3. Overview of the 75 putative OMP families (y1-y75) clustered with CD hit

Y	*E. coli*					*C. coli*			*C. jejuni*			*H. acinonychis*				*H. ailurogastricus*				*H. baculiformis*	*H. hepaticus*	*H. bizzozeronii*				*H. cetorum*		*H. cinaedi*	*H. cynogastricus*	*H. equorum*	*H. felis*				*H. heilmannii*					*H. mustelae*	*H. pylori*						*H. salomonis*			*H. suis*				*H. trogontum*
	536	IAI1	IAI39	K12-W3110	TW14359	15-157360	76339	N29710	00-2425	4031	M1	Hacino1	Hacino2	Hacino3	Hacino4	ASB7	ASB9	ASB11	ASB13	M50	ATC 51449	10	14	CIII	M7	MIT 00-7128	MIT 99-5656	BAA_847	JKM4	eqF1	CS1	CS6	CS7	DS1	ASB1	ASB2	ASB3	ASB6	ASB14	ATC12198	Puno120	G27	F30	J99	India7	SouthAfrica7	M45	R1053	KokIII	HS2	HS4	HS7	HS9	R3554
	NC_008253	NC_011741	NC_011750	NC_007779	NC_013008	NC_022660	NC_022132	NC_022347	NC_022362	NC_022529	NC_017280	FZMD00000000	FZLX00000000	NC_008229	FZLX00000000	CDMG00000000	CDMN00000000	CDML00000000	CDMH00000000	FZMF00000000	NC_004917	FZEH00000000	FZMK00000000	NC_015674	FZLK00000000	NC_017737	NC_017735	NC_020555	FZMQ00000000	FZPO00000000	NC_014810	FZKM00000000	FZKX00000000	FZNI00000000	CDMK00000000	CDMP00000000	CDMI00000000	CDMM00000000	CDMI00000000	NC_013949	NC_017378	NC_011333	NC_017365	NC_000921	NC_017372	NC_022130	FZLZ00000000	OANQ00000000	FZMA00000000	FZLI00000000	FZKI00000000	FZKH00000000	FZLE00000000	FZNG00000000
1																																								x														
2																																								x*														
3	x				x																																																	
4							x																																															x
5																																								x														
6																																			x	x	x		x	x														
7																				x																		x																
8	x	x	x																																																			
9																																								x														
10	x	x	x		x																																																	
11																										x	x*																											
12																															x			x																				
13	x		x																																																			
14												x	x		x											x																												

Y			15	16	17	18	19	20	21	22	23	24	25	26	27	28	29
H. trogontum	R3554	FZNG00000000															
H. suis	HS9	FZLE00000000				x						x					x
	HS7	FZKH00000000										x					x
	HS4	FZKI00000000															x
	HS2	FZLI00000000										x					x
H. salomonis	KokIII	FZMA00000000									x						
	R1053	OANQ00000000															
	M45	FZLZ00000000															
H. pylori	SouthAfrica7	NC_022130														x	
	India7	NC_017372														x	
	J99	NC_000921														x	
	F30	NC_017365														x	
	G27	NC_011333														x	
	Puno120	NC_017378				x										x	
H. mustelae	ATC12198	NC_013949															
H. heilmannii	ASB14	CDMI00000000	x					x	x								
	ASB6	CDMM00000000	x		x				x								
	ASB3	CDMJ00000000	x						x								
	ASB2	CDMP00000000	x					x	x								
	ASB1	CDMK00000000	x						x								
H. felis	DS1	FZNI00000000	x														
	CS7	FZKX00000000															
	CS6	FZKM00000000															
	CS1	NC_014810															
H. equorum	eqF1	FZPO00000000															
H. cynogastricus	JKM4	FZMQ00000000															
H. cinaedi	BAA_847	NC_020555													x		
H. cetorum	MIT 99-5656	NC_017735		x										x		x	
	MIT 00-7128	NC_017737		x							x					x	
H. bizzozeronii	M7	FZLK00000000	*x							x							
	CIII	NC_015674								x							
	14	FZMK00000000	x							x							
	10	FZEH00000000	x			x				x							
H. hepaticus	ATC 51449	NC_004917															
H. baculiformis	M50	FZMF00000000					*x										
H. ailurogastricus	ASB13	CDMH00000000			x			x									
	ASB11	CDML00000000			*x			x									
	ASB9	CDMN00000000			x			x									
	ASB7	CDMG00000000			x			x									
H. acinonychis	Hacino4	FZLX00000000														x	
	Hacino3	NC_008229														x	
	Hacino2	FZLX00000000														x	
	Hacino1	FZMD00000000		x												x	
C. jejuni	M1	NC_017280															
	4031	NC_022529															
	00-2425	NC_022362															
C. coli	N29710	NC_022347															
	76339	NC_022132															
	15-157360	NC_022660															
E. coli	TW14359	NC_013008															
	K12-W3110	NC_007779										x					
	IAI39	NC_011750															
	IAI1	NC_011741															
	536	NC_008253															

Y			30	31	32	33	34	35	36	37	38	39	40	41	42	43	44
H. trogontum	R3554	FZNG00000000															
H. suis	HS9	FZLE00000000															
	HS7	FZKH00000000															x
	HS4	FZKI00000000															
	HS2	FZLI00000000															
H. salomonis	KokIII	FZMA00000000															
	R1053	OANQ00000000															
	M45	FZLZ00000000															
H. pylori	SouthAfrica7	NC_022130															
	India7	NC_017372															
	J99	NC_000921															
	F30	NC_017365															
	G27	NC_011333															
	Puno120	NC_017378															
H. mustelae	ATC12198	NC_013949															
H. heilmannii	ASB14	CDMI00000000												x			
	ASB6	CDMM00000000															
	ASB3	CDMJ00000000															
	ASB2	CDMP00000000															
	ASB1	CDMK00000000															
H. felis	DS1	FZNI00000000															
	CS7	FZKX00000000			x												
	CS6	FZKM00000000			x												
	CS1	NC_014810															
H. equorum	eqF1	FZPO00000000															
H. cynogastricus	JKM4	FZMQ00000000															
H. cinaedi	BAA_847	NC_020555															
H. cetorum	MIT 99-5656	NC_017735															
	MIT 00-7128	NC_017737															
H. bizzozeronii	M7	FZLK00000000															
	CIII	NC_015674				x											
	14	FZMK00000000															
	10	FZEH00000000						x									
H. hepaticus	ATC 51449	NC_004917															
H. baculiformis	M50	FZMF00000000						x									
H. ailurogastricus	ASB13	CDMH00000000															
	ASB11	CDML00000000															
	ASB9	CDMN00000000															
	ASB7	CDMG00000000															
H. acinonychis	Hacino4	FZLX00000000					x					x	*x		x		
	Hacino3	NC_008229					x					x	*x		x		
	Hacino2	FZLX00000000					x					*x	x		x		
	Hacino1	FZMD00000000					x					x	*x		x		
C. jejuni	M1	NC_017280															
	4031	NC_022529															
	00-2425	NC_022362															
C. coli	N29710	NC_022347							x	x						x	
	76339	NC_022132															
	15-157360	NC_022660															
E. coli	TW14359	NC_013008	x	x							x						
	K12-W3110	NC_007779															
	IAI39	NC_011750															
	IAI1	NC_011741															
	536	NC_008253															

H. trogontum	R3554	FZNG00000000							x					x			
H. suis	HS9	FZLE00000000															
	HS7	FZKH00000000															
	HS4	FZKI00000000															
	HS2	FZLI00000000															
H. salomonis	KokIII	FZMA00000000															
	R1053	OANQ00000000															
	M45	FZLZ00000000															
H. pylori	SouthAfrica7	NC_022130															
	India7	NC_017372															
	J99	NC_000921															
	F30	NC_017365															
	G27	NC_011333															
	Puno120	NC_017378															
H. mustelae	ATC12198	NC_013949															
H. heilmannii	ASB14	CDMI00000000											x				
	ASB6	CDMM00000000											x				
	ASB3	CDMJ00000000								x			x				
	ASB2	CDMP00000000											x				
	ASB1	CDMK00000000											x				
H. felis	DS1	FZNI00000000															
	CS7	FZKX00000000															
	CS6	FZKM00000000															
	CS1	NC_014810															
H. equorum	eqF1	FZPO00000000															
H. cynogastricus	JKM4	FZMQ00000000															
H. cinaedi	BAA_847	NC_020555															
H. cetorum	MIT 99-5656	NC_017735															
	MIT 00-7128	NC_017737															
H. bizzozeronii	M7	FZLK00000000			x		x								x		
	CIII	NC_015674															
	14	FZMK00000000			x												
	10	FZEH00000000					x								x		
H. hepaticus	ATC 51449	NC_004917															
H. baculiformis	M50	FZMF00000000															
H. ailurogastricus	ASB13	CDMH00000000															
	ASB11	CDML00000000															
	ASB9	CDMN00000000															
	ASB7	CDMG00000000															
H. acinonychis	Hacino4	FZLX00000000		x	x	x						x			x		x
	Hacino3	NC_008229		x	x	x						x			x		*x
	Hacino2	FZLX00000000		x	x	x						x					x
	Hacino1	FZMD00000000		x	x	x						x			x		x
C. jejuni	M1	NC_017280															
	4031	NC_022529												x			
	00-2425	NC_022362												x			
C. coli	N29710	NC_022347															
	76339	NC_022132															
	15-157360	NC_022660															
E. coli	TW14359	NC_013008															
	K12-W3110	NC_007779															
	IAI39	NC_011750															
	IAI1	NC_011741															
	536	NC_008253	x					x			x						
Y			45	46	47	48	49	50	51	52	53	54	55	56	57	58	59

H. trogontum	R3554	FZNG00000000															
H. suis	HS9	FZLE00000000										x					
	HS7	FZKH00000000										x					
	HS4	FZKI00000000										x					
	HS2	FZLI00000000										x					
H. salomonis	KokIII	FZMA00000000											x				
	R1053	OANQ00000000											x				
	M45	FZLZ00000000											x				
H. pylori	SouthAfrica7	NC_022130															
	India7	NC_017372															
	J99	NC_000921															
	F30	NC_017365															
	G27	NC_011333															
	Puno120	NC_017378															
H. mustelae	ATC12198	NC_013949															
H. heilmannii	ASB14	CDMI00000000															
	ASB6	CDMM00000000															
	ASB3	CDMJ00000000															
	ASB2	CDMP00000000															
	ASB1	CDMK00000000															
H. felis	DS1	FZNI00000000															
	CS7	FZKX00000000														x	
	CS6	FZKM00000000															
	CS1	NC_014810															
H. equorum	eqF1	FZPO00000000															
H. cynogastricus	JKM4	FZMQ00000000														x	
H. cinaedi	BAA_847	NC_020555															
H. cetorum	MIT 99-5656	NC_017735					x										
	MIT 00-7128	NC_017737					x										
H. bizzozeronii	M7	FZLK00000000											x				
	CIII	NC_015674															
	14	FZMK00000000															
	10	FZEH00000000											*x				
H. hepaticus	ATC 51449	NC_004917															
H. baculiformis	M50	FZMF00000000															
H. ailurogastricus	ASB13	CDMH00000000															
	ASB11	CDML00000000															
	ASB9	CDMN00000000															
	ASB7	CDMG00000000															
H. acinonychis	Hacino4	FZLX00000000	x	x	x			x			x			x	x		x
	Hacino3	NC_008229	*x	x	x									x	*x		
	Hacino2	FZLX00000000	x	x	x						x			x	x		x
	Hacino1	FZMD00000000	*x	x	x			x			x				x		
C. jejuni	M1	NC_017280							x								
	4031	NC_022529							x								
	00-2425	NC_022362							x								
C. coli	N29710	NC_022347															
	76339	NC_022132															
	15-157360	NC_022660															
E. coli	TW14359	NC_013008															
	K12-W3110	NC_007779															
	IAI39	NC_011750								x							
	IAI1	NC_011741				x											
	536	NC_008253				x				x							
Y			60	61	62	63	64	65	66	67	68	69	70	71	72	73	74

75																																																						x*

Shown are the strain names of *E. coli* and the *Campylobacter* and *Helicobacter* species with homologous putative OMP sequences. Strains with multiple OMP sequences belonging to the same family are indicated with an asterisk *. “x” indicates that the protein is present. In general, the CD-HIT Y-families were mostly found in only one species and often not in all analyzed strains.

Table S4. Subgroups of family 42 – the Outer membrane factor (OMF) family

Sub-groups of family 42 the OMF family	*E. coli*					*C. coli*			*C. jejuni*			*H. acinonychis*				*H. ailurogastricus*				*H. baculiformis*	*H. hepaticus*	*H. bizzozeronii*				*H. cetorum*		*H. cinaedi*	*H. cynogastricus*	*H. equorum*	*H. felis*				*H. heilmannii*					*H. mustelae*	*H. pylori*						*H. salomonis*			*H. suis*				*H. trogontum*
	536	IAI1	IAI39	K12-W3110	TW14359	15-157360	76339	N29710	00-2425	4031	M1	Hacino1	Hacino2	Hacino3	Hacino4	ASB7	ASB9	ASB11	ASB13	M50	ATC 51449	10	14	CIII	M7	MIT 00-7128	MIT 99-5656	BAA_847	JKM4	eqF1	CS1	CS6	CS7	DS1	ASB1	ASB2	ASB3	ASB6	ASB14	ATC12198	Puno120	G27	F30	J99	India7	SouthAfrica7	M45	R1053	KokIII	HS2	HS4	HS7	HS9	R3554
	NC_008253	NC_011741	NC_011750	NC_007779	NC_013008	NC_022660	NC_022132	NC_022347	NC_022362	NC_022529	NC_017280	FZMD00000000	FZLX00000000	NC_008229	FZLX00000000	CDMG00000000	CDMN00000000	CDML00000000	CDMH00000000	FZMF00000000	NC_004917	FZEH00000000	FZMK00000000	NC_015674	FZLK00000000	NC_017737	NC_017735	NC_020555	FZMQ00000000	FZPO00000000	NC_014810	FZKM00000000	FZKX00000000	FZNI00000000	CDMK00000000	CDMP00000000	CDMJ00000000	CDMM00000000	CDMI00000000	NC_013949	NC_017378	NC_011333	NC_017365	NC_000921	NC_017372	NC_022130	FZLZ00000000	OANQ00000000	FZMA00000000	FZLI00000000	FZKI00000000	FZKH00000000	FZLE00000000	FZNG00000000
OMEP/Tr	x		x		x	x	x	x	x	x	x					x	x	x	x	x	x	x	x	x	x			x	x		x	x	x	x	x	x	x	x	x	x							x	x	x	x	x	x	x	
lipases												x	x	x	x							x	x	x	x	x	x														x	x	x	x	x	x				x	x	x	x	
HMP												x	x	x	x	x	x	x	x	x		x	x	x	x	x	x		x	x	x	x	x	x	x	x	x	x	x	x	x	x	x	x	x	x	x	x	x	x	x	x	x	x
CrdB																							x								x	x	x	x						x							x	x	x					
RNDTr /EP	x	x	x	x	x	x	x	x	x	x	x					x	x	x	x		x							x		x					x	x	x	x	x															
ABC TrPe																					x							x		x																								x
CoTr/EP																					x							x		x																								x
TolC	x	x	x	x	x																																																	
MdtQ	x	x	x	x	x																																																	
CusC	x	x	x	x	x																																																	
FimD		x	x	x	x																																																	

"x" indicates that the protein is present. **OMEP/Tr** = Outer membrane efflux proteins/transporters; **HMP** = Hypothetical membrane proteins; **CrdB** = Copper resistance determinant proteins Crdb; **RNDTr/EP** = RND transporters/efflux proteins; **ABC TrPe** = ABC transporter permeases; **CoTr/EP** = (nickel) cobalt transporters/efflux proteins; **TolC** = Outer membrane channel proteins TolC; **MdtQ** = Multidrug resistance outer membrane proteins; **CusC** = Cation transporters/efflux systems; **FimD** = Outer membrane usher proteins FimD

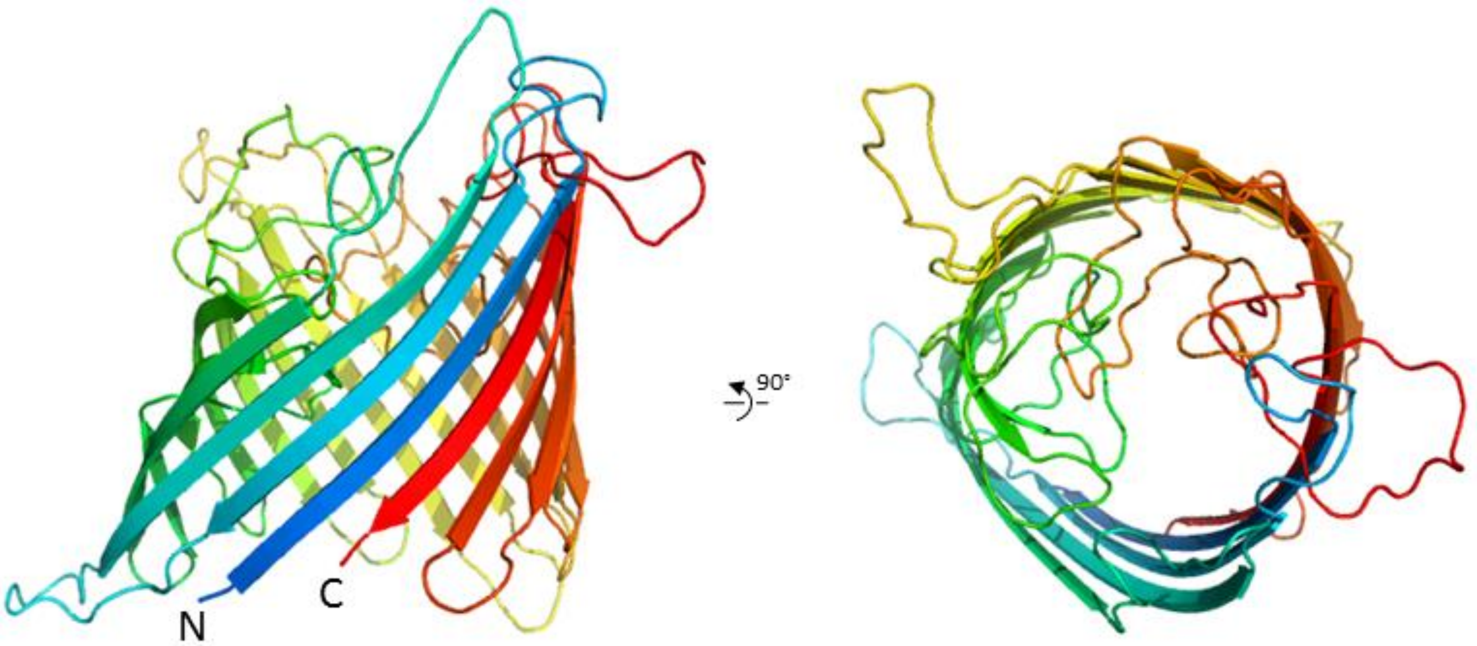

Figure S1. Ribbon diagram of 3D threaded model of *H. pylori* HofA. Remote homology searches point to a structural homology the Hof family proteins with 18-stranded porins of the Occ family. The displayed model is that of HofA (seq ID: Hheil1_OMP1507_gastric) homology modeled to *P. aeruginosa* OpdQ (PDB ID: 3R24; ref DOI: 10.1371/journal.pbio.1001242) using RaptorX (Ma et al. 2013). The ribbon diagram shows residues 65 to 465, colored blue to red from N- to C-terminus.

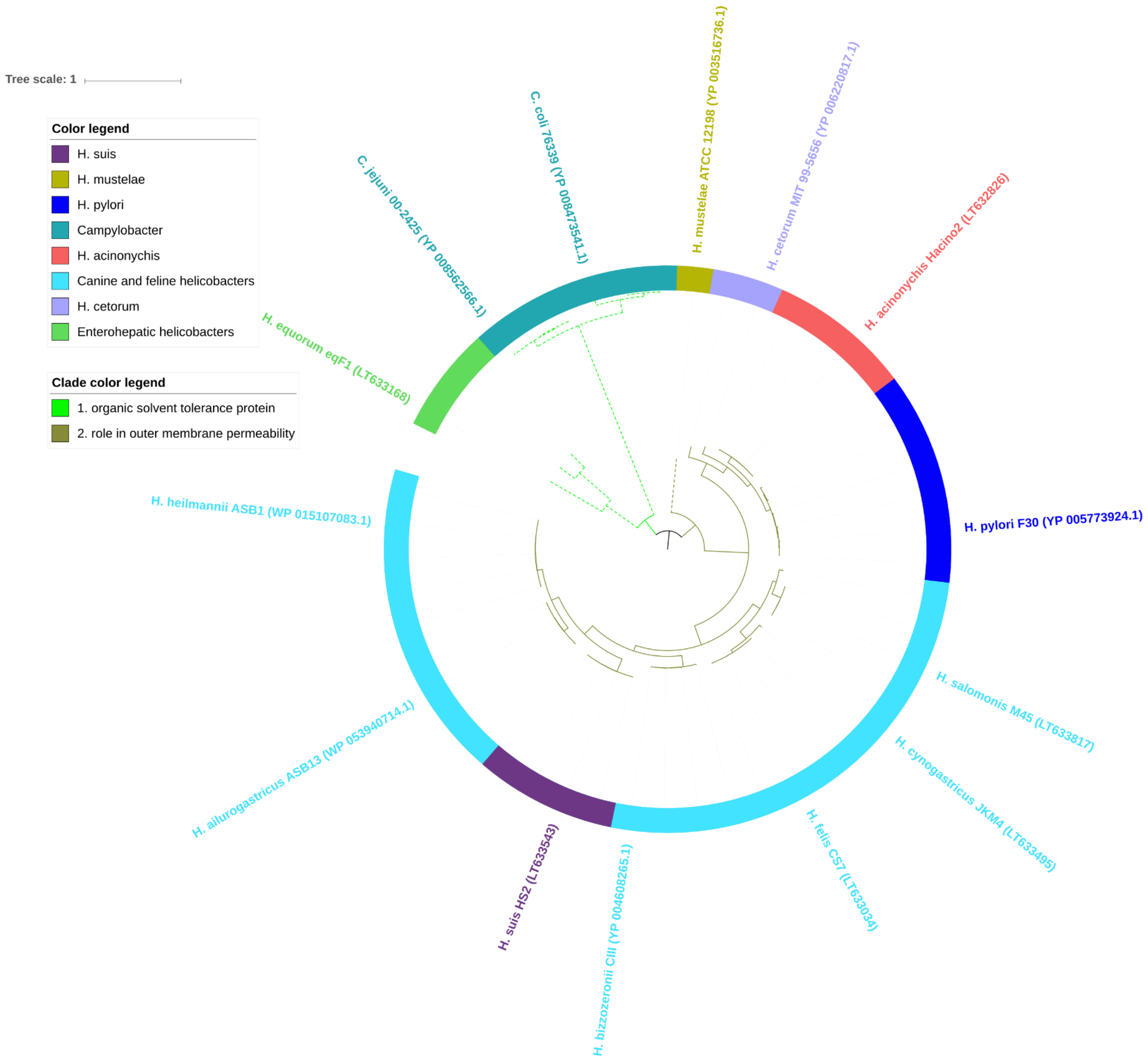

Tree scale: 1
Color legend
H. suis
H. mustelae
H. pylori
Campylobacter
H. acinonychis
Canine and feline helicobacters
H. cetorum
Enterohepatic helicobacters
Clade color legend
1. organic solvent tolerance protein
2. role in outer membrane permeability
C. jejuni 00-2425 (YP 008562566.1)
C. coli 76339 (YP 008473541.1)
H. mustelae ATCC 12198 (YP 003516736.1)
H. cetorum MIT 99-5656 (YP 006220817.1)
H. acinonychis Hacino2 (LT632826)
H. equorum eqF1 (LT633168)
H. pylori F30 (YP 005773924.1)
H. heilmannii ASB1 (WP 015107083.1)
H. salomonis M45 (LT633817)
H. cynogastricus JKM4 (LT633495)
H. ailurogastricus ASB13 (WP 053940714.1)
H. felis CS7 (LT633034)
H. suis HS2 (LT633543)
H. bizzozeronii CIII (YP 004608265.1)

Figure S2. Phylogenetic tree of Family 14 – Increased membrane permeability (Imp) or organic solvent tolerance (OstA) - Imp/OstA. One orthologous OMP is present in each of the examined strains of *Campylobacter* and *Helicobacter*, except for *H. trogontum*, and no orthologous OMPs are detected in *E. coli*. The Imp/OstA OMP of *H. mustelae* (yellow-green) clusters in between those of gastric and enterohepatic helicobacters (green). OMPs of *H. mustelae*, enterohepatic helicobacters and *Campylobacter* species are indicated by dashed clade lines. Per clade, minimum one accession number is added.

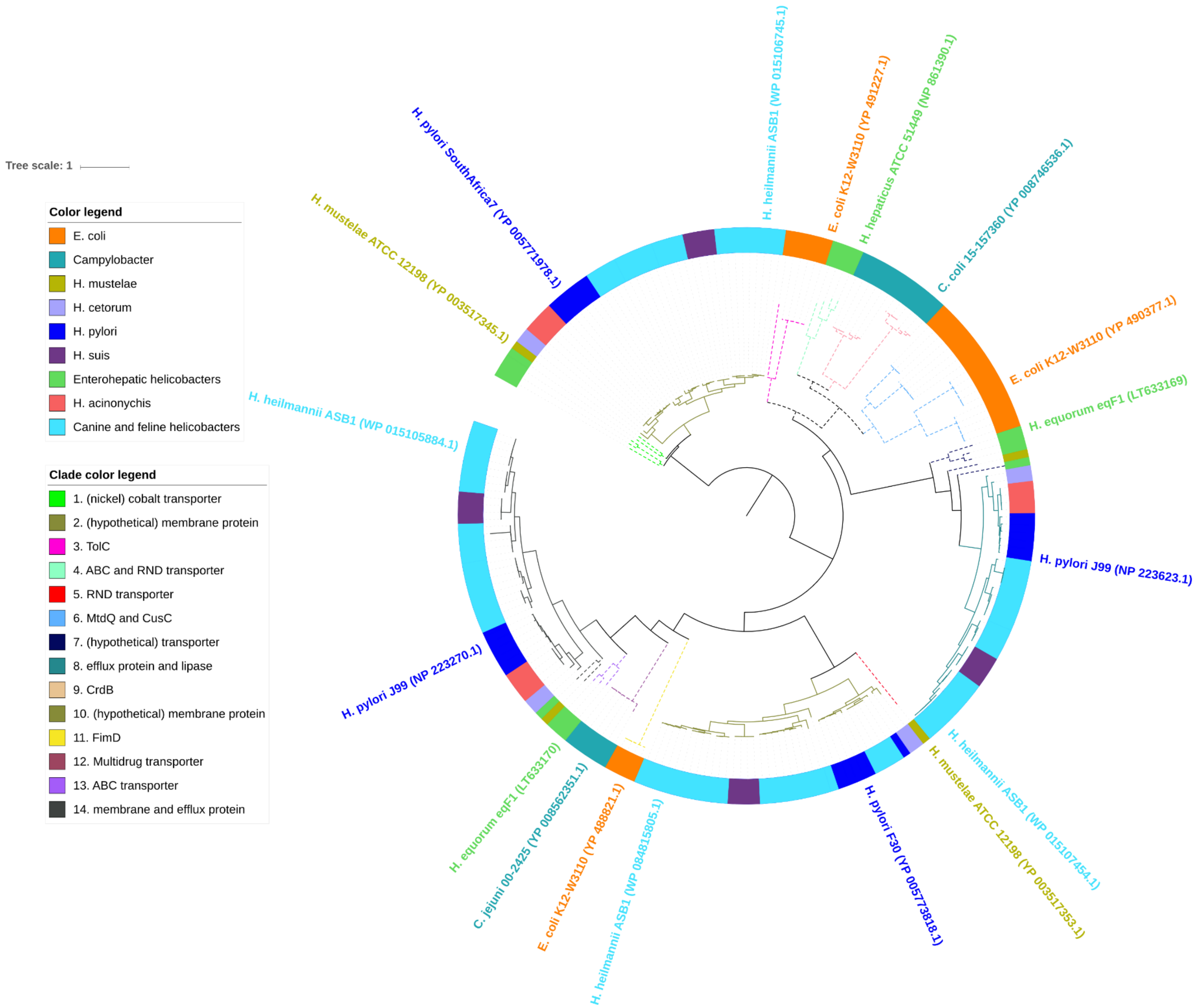
Tree scale: 1
Color legend
E. coli
Campylobacter
H. mustelae
H. cetorum
H. pylori
H. suis
Enterohepatic helicobacters
H. acinonychis
Canine and feline helicobacters
Clade color legend
1. (nickel) cobalt transporter
2. (hypothetical) membrane protein
3. TolC
4. ABC and RND transporter
5. RND transporter
6. MtdQ and CusC
7. (hypothetical) transporter
8. efflux protein and lipase
9. CrdB
10. (hypothetical) membrane protein
11. FimD
12. Multidrug transporter
13. ABC transporter
14. membrane and efflux protein
H. heilmannii ASB1 (WP 015106745.1)
E. coli K12-W3110 (YP 491227.1)
H. hepaticus ATCC 51449 (NP 861390.1)
C. coli 15-157360 (YP 008746536.1)
E. coli K12-W3110 (YP 490377.1)
H. equorum eqF1 (LT633169)
H. pylori J99 (NP 223623.1)
H. heilmannii ASB1 (WP 015107454.1)
H. mustelae ATCC 12198 (YP 003517353.1)
H. pylori F30 (YP 005773818.1)
H. heilmannii ASB1 (WP 084815805.1)
E. coli K12-W3110 (YP 488821.1)
C. jejuni 00-2425 (YP 008562351.1)
H. equorum eqF1 (LT633170)
H. pylori J99 (NP 223270.1)
H. heilmannii ASB1 (WP 015105884.1)
H. mustelae ATCC 12198 (YP 003517345.1)
H. pylori SouthAfrica7 (YP 005771978.1)

Figure S3. Phylogenetic tree of Family 42 – Outer membrane factor (OMF). Orthologous OMPs are present in all examined strains of *E. coli, Campylobacter* and *Helicobacter*. Several different subgroups are indicated. OMPs of *H. mustelae* (yellow-green) cluster together with enterohepatic helicobacters (green). OMPs of *H. mustelae*, enterohepatic helicobacters and *Campylobacter* species are indicated by dashed clade lines. Per clade, minimum one accession number is added.

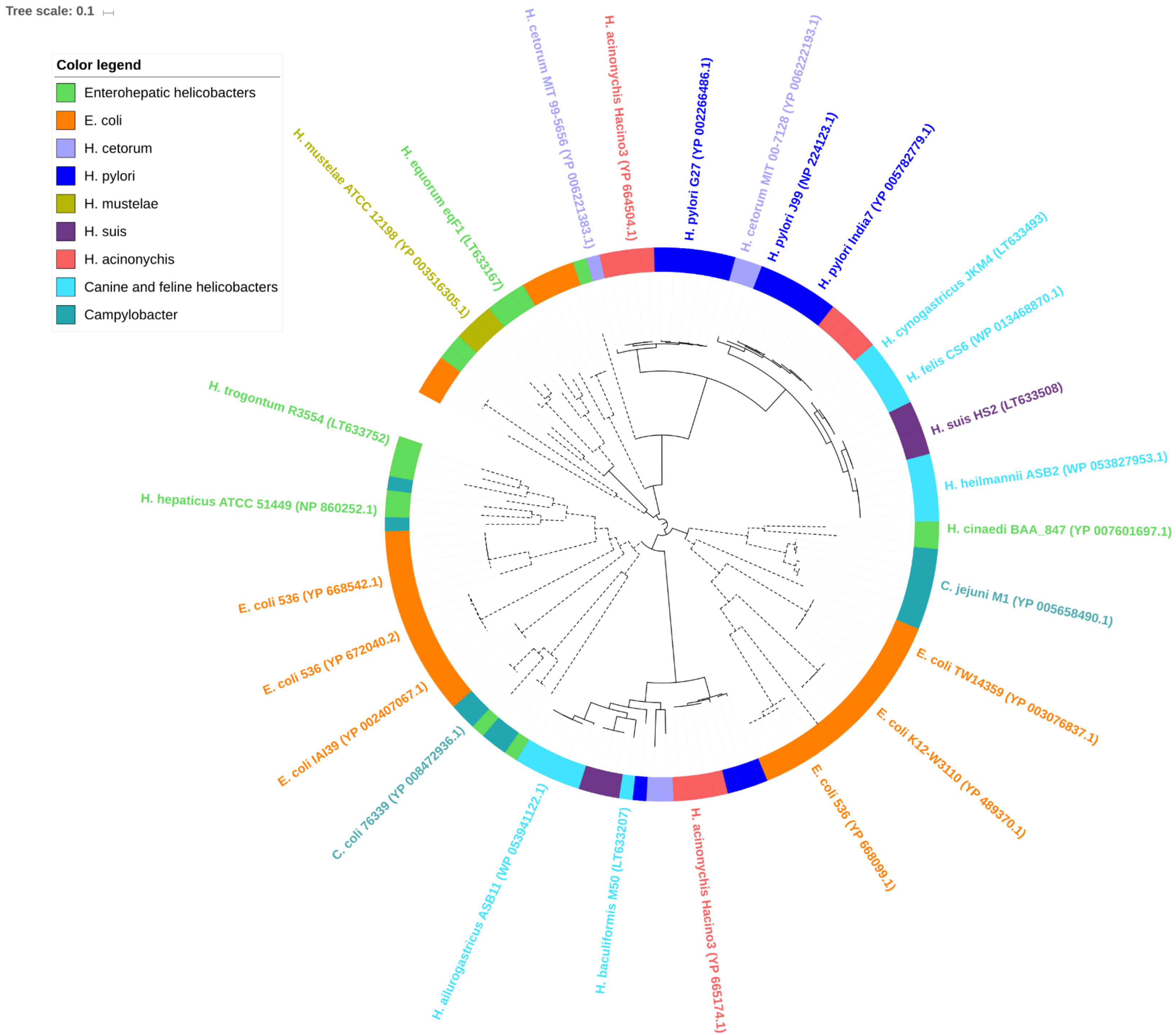

Tree scale: 0.1
Color legend
Enterohepatic helicobacters
E. coli
H. cetorum
H. pylori
H. mustelae
H. suis
H. acinonychis
Canine and feline helicobacters
Campylobacter
H. cetorum MIT 99-5656 (YP 006221383.1)
H. acinonychis Hacino3 (YP 664504.1)
H. pylori G27 (YP 002266486.1)
H. cetorum MIT 00-7128 (YP 006222193.1)
H. pylori J99 (NP 224123.1)
H. pylori India7 (YP 005782779.1)
H. cynogastricus JKM4 (LT633493)
H. felis CS6 (WP 013468870.1)
H. suis HS2 (LT633508)
H. heilmannii ASB2 (WP 053827953.1)
H. cinaedi BAA_847 (YP 007601697.1)
C. jejuni M1 (YP 005658490.1)
E. coli TW14359 (YP 003076837.1)
E. coli K12-W3110 (YP 489370.1)
E. coli 536 (YP 668099.1)
H. acinonychis Hacino3 (YP 665174.1)
H. baculiformis M50 (LT633207)
H. ailurogastricus ASB11 (WP 053941122.1)
C. coli 76339 (YP 008472936.1)
E. coli IAI39 (YP 002407067.1)
E. coli 536 (YP 672040.2)
E. coli 536 (YP 668542.1)
H. hepaticus ATCC 51449 (NP 860252.1)
H. trogontum R3554 (LT633752)
H. mustelae ATCC 12198 (YP 003516305.1)
H. equorum eqF1 (LT633167)

Figure S4. Phylogenetic tree of Family 3 the outer membrane TonB dependent receptors. Orthologous OMPs are present in all examined strains of *E. coli, Campylobacter* and Helicobacter except for *H. bizzozeronii* and *H. salomonis*. OMPs of *H. mustelae* (yellow-green) cluster together with those of enterohepatic helicobacters (green). OMPs of enterohepatic helicobacters (green) cluster together with those of *Campylobacter* (dark cyan). OMPs of *H. mustelae*, enterohepatic helicobacters and *Campylobacter* species are indicated by dashed clade lines. Per clade, minimum one accession number is added.

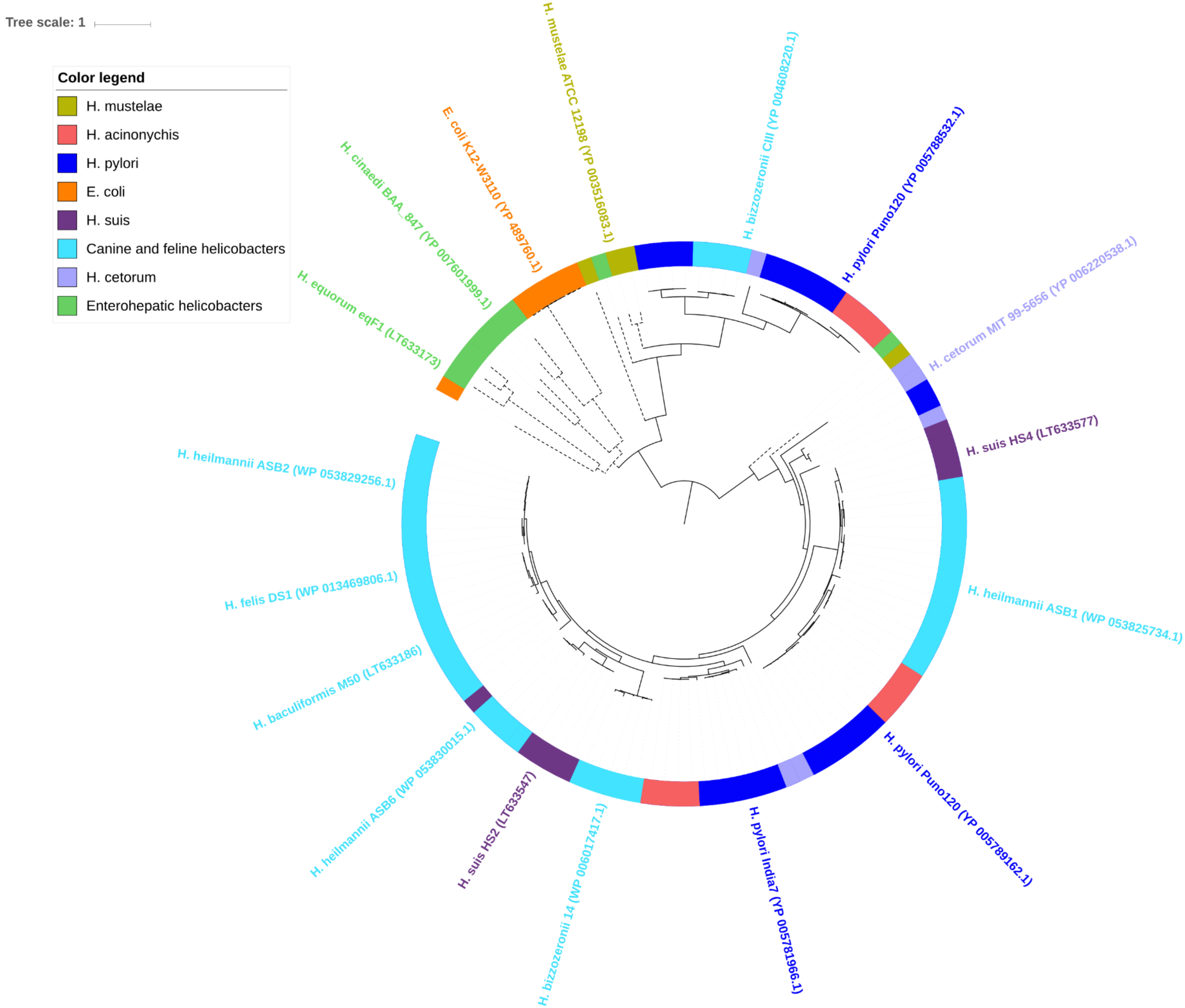

Tree scale: 1
Color legend
H. mustelae
H. acinonychis
H. pylori
E. coli
H. suis
Canine and feline helicobacters
H. cetorum
Enterohepatic helicobacters
H. mustelae ATCC 12198 (YP 003516083.1)
E. coli K12-W3110 (YP 489760.1)
H. cinaedi BAA_847 (YP 007601999.1)
H. equorum eqF1 (LT633173)
H. bizzozeronii CIII (YP 004608220.1)
H. pylori Puno120 (YP 005788532.1)
H. cetorum MIT 99-5656 (YP 006220538.1)
H. suis HS4 (LT633577)
H. heilmannii ASB1 (WP 053825734.1)
H. pylori Puno120 (YP 005789162.1)
H. pylori India7 (YP 005781966.1)
H. bizzozeronii 14 (WP 006017417.1)
H. suis HS2 (LT633547)
H. heilmannii ASB6 (WP 053830015.1)
H. baculiformis M50 (LT633186)
H. felis DS1 (WP 013469806.1)
H. heilmannii ASB2 (WP 053829256.1)

Figure S5. **Phylogenetic tree of Family X2 TonB dependent receptor plug domains.** Orthologous OMPs are present in all examined strains of *E. coli* and *Helicobacter* except for *H. salomonis*, and are absent in *C. coli* and *C. jejuni*. OMPs from *H. mustelae* (yellow-green) clustered in between those of gastric and enterohepatic (green) helicobacters. OMPs of *H. mustelae* and enterohepatic helicobacters are indicated by dashed clade lines. Per clade, minimum one accession number is added.

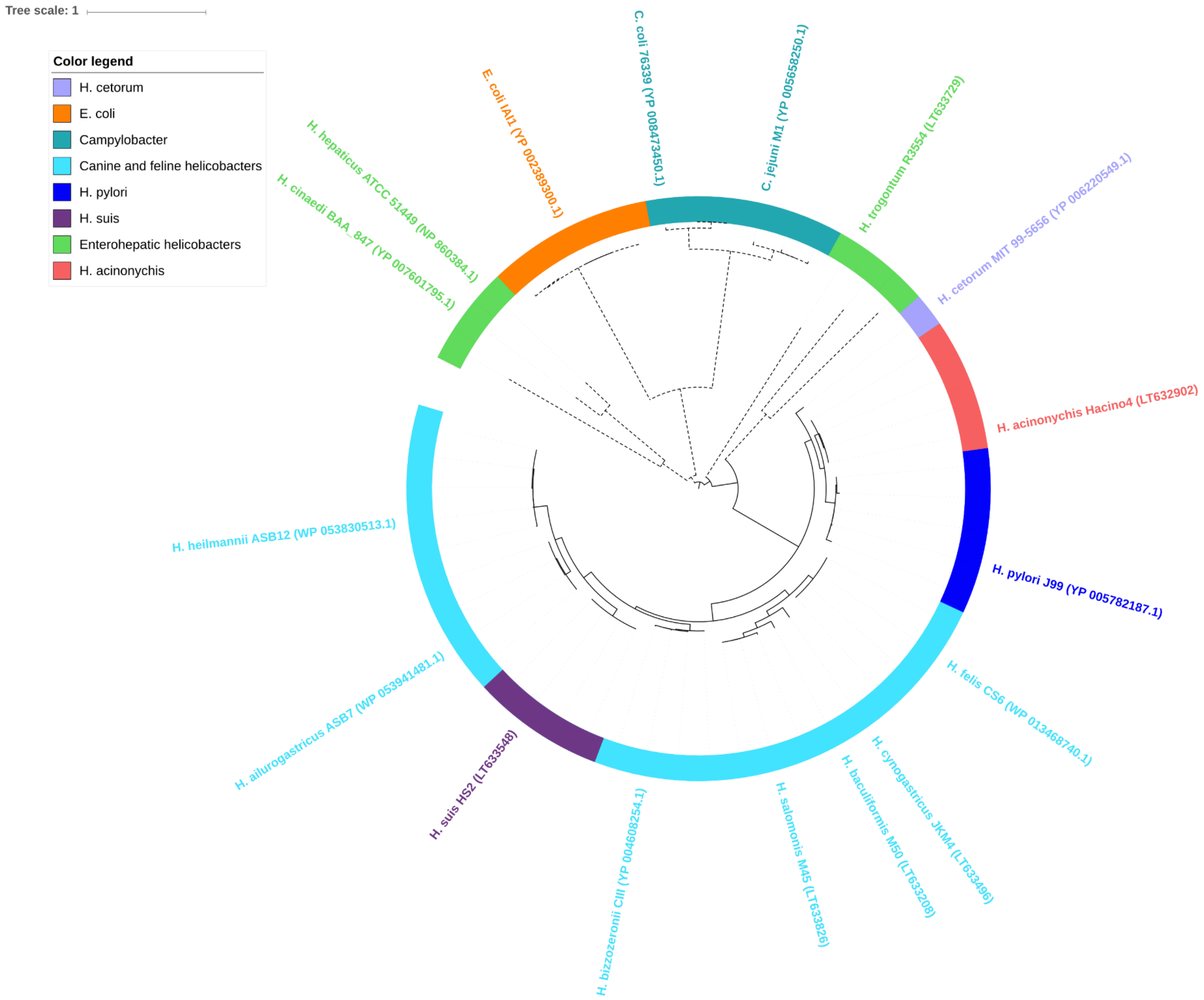

Tree scale: 1
Color legend
H. cetorum
E. coli
Campylobacter
Canine and feline helicobacters
H. pylori
H. suis
Enterohepatic helicobacters
H. acinonychis
C. coli 76339 (YP 008473450.1)
C. jejuni M1 (YP 005658250.1)
H. trogontum R3554 (LT633729)
H. cetorum MIT 99-5656 (YP 006220549.1)
H. acinonychis Hacino4 (LT632902)
H. pylori J99 (YP 005782187.1)
H. felis CS6 (WP 013468740.1)
H. cynogastricus JKM4 (LT633496)
H. baculiformis M50 (LT633208)
H. salomonis M45 (LT633826)
H. bizzozeronii CIII (YP 004608254.1)
H. suis HS2 (LT633548)
H. ailurogastricus ASB7 (WP 053941481.1)
H. heilmannii ASB12 (WP 053830513.1)
H. cinaedi BAA_847 (YP 007601795.1)
H. hepaticus ATCC 51449 (NP 860384.1)
E. coli IAI1 (YP 002389300.1)

Figure S6. Phylogenetic tree of Family 38 – Outer membrane phospholipase (OMPLA). One orthologous OMP is present in each of the examined strains of *E. coli, Campylobacter* and *Helicobacter* except for *H. mustelae, H. equorum, H. cetorum* MIT 00-7128 and *H. pylori* G27, in which this OMP is absent. In *H. trogontum*, 4 OMPLA OMPs are present. OMPs of enterohepatic helicobacters and *Campylobacter* species are indicated by dashed clade lines. Per clade, minimum one accession number is added.
